# *In* *silico* design and evaluation of a multi-epitope vaccine targeting eyach virus for the prevention of tick-borne encephalitis in humans

**DOI:** 10.1038/s41598-026-59953-2

**Published:** 2026-06-30

**Authors:** Waad A. Aljohani, Masood Alam Khan, Fawzyah Obeedallah Albaldi, Alaa Karkashan, Khulud Bukhari, Sarah Nasser Alnuwaysir, Razan Abdalrahman Almohimid, Amal M. H. Mackawy, Amal H. Mohammed, Khaled S. Allemailem

**Affiliations:** 1 Department of Basic Health Sciences, College of Applied Medical Sciences, Qassim University, Buraydah, 51452 Saudi Arabia; 2 Department of Biology, Faculty of Science, Al-Baha University, Alaqiq, 65779-7738 Saudi Arabia; 3 Department of Biological Sciences, College of Science, University of Jeddah, Jeddah, 21959 Saudi Arabia; 4 Department of Microbiology and Parasitology, College of Veterinary Medicine, King Faisal University, Hofuf, Al-Ahsa, 36362 Saudi Arabia; 5 Department of Medical Laboratories, College of Applied Medical Sciences, Qassim University, Buraydah, 51452 Saudi Arabia

**Keywords:** Multi-epitope vaccine, Eyach virus, Molecular docking, Molecular dynamics simulation, Immune simulation, Epitope prediction, Vaccine design, Toll-like receptors, Computational biology and bioinformatics, Drug discovery, Immunology, Microbiology

## Abstract

Eyach virus is a tick-borne pathogen associated with neurological complications resembling encephalitis, and its increasing emergence highlights a growing public health concern. The absence of specific antiviral therapies and limited surveillance data emphasize the urgent need for effective preventive strategies such as vaccine development. This study presents an immunoinformatics-driven design and in silico evaluation of a multi-epitope vaccine candidate targeting Eyach virus. Structural proteins VP5 and VP7 were analyzed to identify highly antigenic, non-allergenic, and non-toxic B-cell, CTL, and HTL epitopes. The selected epitopes demonstrated broad global population coverage of 97.94%, indicating wide immunogenic applicability. These epitopes were assembled into a 256 amino acid vaccine construct using suitable linkers and β-defensin-3 as an adjuvant. Physicochemical properties analysis revealed a stable, hydrophilic, and soluble protein profile. Structural modeling and refinement confirmed high stereochemical quality, with 96.3% of residues located in favored regions. Molecular docking analysis indicated favorable predicted interactions between the vaccine construct and TLR3 and TLR4. Molecular dynamics simulations further confirmed the structural stability, compactness, and consistent behavior of the complexes. In addition, MM/GBSA analysis revealed favorable binding free energies, with the vaccine–TLR3 complex exhibiting a stronger binding free energy of − 203.29 kcal/mol. Immune simulation predicted robust humoral and cellular immune responses, including elevated immunoglobulin levels, cytokine production, and memory cell formation following a three-dose regimen. Overall, the findings suggest that the proposed multi-epitope vaccine is a promising candidate against Eyach virus; however, experimental validation is required to confirm its safety and efficacy.

## Introduction

Eyach virus is an arthropod-borne virus that belongs to the genus Coltivirus within the family *Spinareoviridae*^1^. It is primarily transmitted through infected ticks, particularly Ixodes species, which serve as the natural vectors of the virus^2^. Infections associated with Eyach virus have been linked to neurological manifestations resembling tick-borne encephalitis and other neuroinvasive diseases^3,4^. The neurotropic nature of Eyach virus suggests its potential to affect the central nervous system and contribute to neurological disease^2^. The limited availability of epidemiological and clinical data on Eyach virus highlights the need for further research to better understand its pathogenicity and public health significance^5^.

Although reported cases of Eyach virus infection remain limited, the virus has been detected in tick populations and remains a recognized tick-borne pathogen of public health interest^6^. Underreporting has also been recognized for related tick-borne viruses, including Tick-borne encephalitis virus, which may contribute to an underestimation of disease burden^7^. In addition, environmental changes that influence tick distribution may increase the risk of human exposure to tick-borne pathogens^8^. These factors highlight the need for improved surveillance and a clearer understanding of the epidemiology of Eyach virus. At present, no specific antiviral therapy has been established for Eyach virus infection, and patient management remains largely supportive^9^. This limitation is particularly relevant in cases involving neurological symptoms. Similar therapeutic challenges have been reported for other tick-borne viral infections, including Tick-borne encephalitis virus, for which treatment options remain limited^10^. The lack of targeted therapies emphasizes the importance of preventive strategies, particularly vaccine development^11^.

Preventive immunization offers an effective means of controlling viral diseases by enabling the immune system to recognize and neutralize pathogens before infection is established^12^. Vaccines function by stimulating both humoral and cellular immune responses in a targeted manner^13^. In recent years, vaccine development has increasingly shifted toward epitope-focused strategies that utilize selected antigenic regions to induce protective immune responses^14^. These multi-epitope vaccine designs allow for precise immune targeting and can be tailored to enhance population-wide coverage^15^. In parallel, advances in computational biology have enabled the use of predictive algorithms and structural modeling tools for the rational design of vaccine candidates with improved accuracy^16^. In silico approaches facilitate the identification of immunogenic regions, evaluation of their compatibility, and assessment of structural stability, thereby streamlining the vaccine development process^17^.

Despite the potential public health significance of Eyach virus, vaccine development efforts against this pathogen remain extremely limited. To the best of our knowledge, no vaccine candidate against Eyach virus has been reported to date. Furthermore, the present study utilizes the recently developed IEDB Next Generation Tools pipeline, including NetMHCpan 4.1 EL and NetMHCIIpan 4.1 EL, together with updated antigenicity and allergenicity prediction platforms, namely VaxiJen v3.0 and AllerTOP v2.1, to improve the reliability of epitope selection. Therefore, this work represents the first comprehensive computational study focused on the identification of immunogenic epitopes and the rational design of a multi-epitope vaccine candidate against Eyach virus.

The present study aimed to develop a multi-epitope vaccine candidate against Eyach virus using a systematic computational approach. The study focused on identifying immunologically relevant regions capable of inducing both B-cell- and T-cell-mediated immune responses and integrating them into a single vaccine construct. The resulting vaccine candidate was further evaluated for its immunogenic potential, safety profile, and structural integrity. In addition, molecular interaction analyses were performed to assess the ability of the construct to engage immune receptors. Through these analyses, the study sought to propose a rationally designed multi-epitope vaccine candidate that could serve as a foundation for future experimental validation and contribute to the development of preventive strategies against Eyach virus infection.

## Materials and methods

**Fig. 1:**
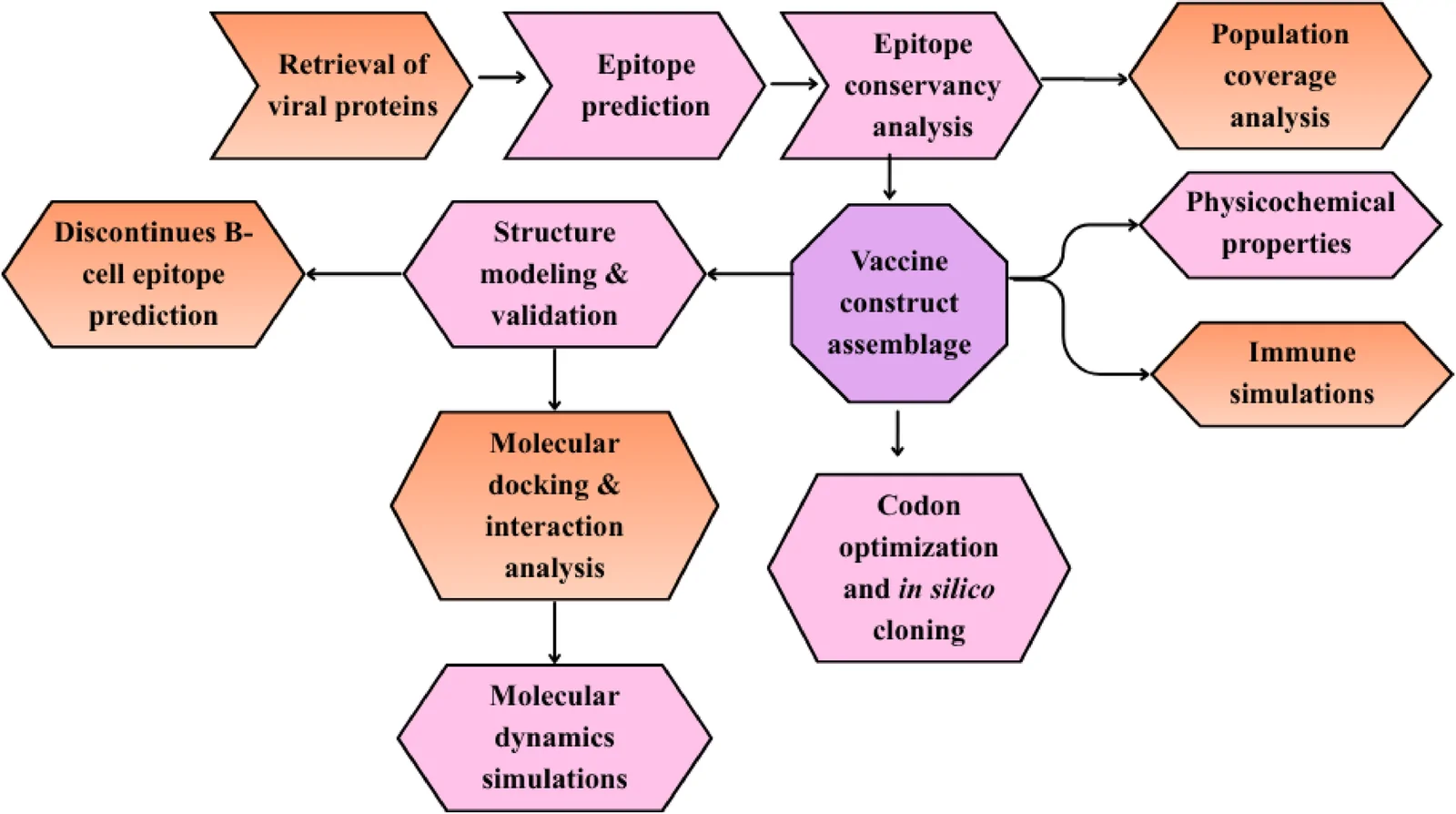
Schematic overview of the computational workflow used for the design and evaluation of the multi-epitope vaccine candidate.

The overall workflow of the present study followed a systematic immunoinformatics pipeline, beginning with the retrieval of viral proteins followed by epitope identification and prediction. The selected epitopes were further analyzed for population coverage and subsequently integrated into a multi-epitope vaccine construct. The construct was evaluated through physicochemical characterization, structural modeling, molecular docking, and molecular dynamics simulations. A schematic representation of the complete methodology is presented in Fig. 1.

### Retrieval of protein

Protein sequences of Eyach virus, including VP5 (NCBI ID: QYV43097.1) and VP7 (NCBI ID: QYV43099.1), were retrieved from the NCBI database (https://www.ncbi.nlm.nih.gov/) in FASTA format. VP5 and VP7 were selected for vaccine design because they are outer capsid proteins involved in viral infectivity, viral entry, and host–virus interactions, making them attractive targets for immune recognition. The transmembrane topology of the selected proteins was analyzed using TMHMM Server v2.0 (https://services.healthtech.dtu.dk/services/TMHMM-2.0/) to identify membrane-spanning regions^18^. The antigenicity of the proteins was evaluated using VaxiJen v3.0 (https://www.ddg-pharmfac.net/vaxijen3/home/) to determine their potential to induce a protective immune response^19^. Allergenicity assessment was performed using AllerTOP v2.1 (https://www.ddg-pharmfac.net/allertop_test/) to ensure the selection of non-allergenic candidates for vaccine design^20^.

### Epitope prediction

Linear B-cell epitopes were predicted using ABCpred, which employs machine learning-based pattern recognition to identify antibody-accessible regions within protein sequences. This approach focuses on detecting short peptide fragments with a high likelihood of surface exposure and immunoreactivity. To identify cytotoxic T-lymphocyte (CTL) epitopes, peptide interactions with human MHC class I molecules were analyzed using NetMHCpan 4.1 EL through the IEDB Next Generation Tools platform (https://nextgen-tools.iedb.org/pipeline? tool=tc1)^21^. Binding affinity was interpreted using percentile ranking, and peptides with a percentile rank of less than 1 were selected. Helper T-lymphocyte (HTL) epitopes were predicted using NetMHCIIpan 4.1 EL (https://nextgen-tools.iedb.org/pipeline? tool=tc2)^22^, which enables the evaluation of peptide binding across a broad range of HLA class II alleles. Candidate epitopes were shortlisted based on percentile ranking, and peptides exhibiting a percentile rank of less than 10 were selected. To refine epitope selection, all predicted B-cell and T-cell epitopes underwent additional screening based on immunological and safety-related characteristics. Their antigenic potential was evaluated using VaxiJen v3.0, while their allergenicity was assessed using AllerTOP v2.1. Furthermore, toxicity profiling was performed using ToxinPred to eliminate potentially harmful peptide candidates. Only epitopes meeting all selection criteria, including high antigenicity, non-allergenicity, and non-toxicity, were retained for subsequent stages of vaccine construction.

### Epitope conservancy analysis

To evaluate the potential applicability of the selected epitopes across different Eyach virus isolates, an epitope conservancy analysis was performed. The amino acid sequences of the VP5 and VP7 proteins were subjected to BLASTp analysis against the NCBI protein database to identify homologous Eyach virus sequences. Due to the limited number of Eyach virus protein sequences currently available in public databases, a total of three VP5 sequences and two VP7 sequences, including the reference sequences used for epitope prediction, were retrieved for analysis. The conservancy of the selected B-cell, CTL, and HTL epitopes was assessed using the Epitope Conservancy Analysis tool available through the Immune Epitope Database (IEDB) (http://tools.iedb.org/conservancy/)^23^. Conservancy was calculated as the percentage of sequences containing an identical epitope sequence.

### Population coverage analysis

The distribution of the selected T-cell epitopes across global human populations was evaluated using the population coverage tool available through the IEDB (http://tools.iedb.org/population/)^24^. This approach estimates the frequency of the associated HLA alleles in different ethnic and geographic populations, thereby predicting the proportion of individuals who are likely to mount an immune response to the selected epitopes.

### Vaccine construct assemblage

The multi-epitope vaccine construct was designed by sequentially fusing the selected B-cell, CTL, and HTL epitopes into a single continuous polypeptide chain. This arrangement was designed to preserve the individual immunological properties of each epitope while supporting appropriate antigen processing within the host immune system. To ensure effective separation between epitopic regions and maintain structural flexibility, specific linker sequences were incorporated. KK linkers were used to connect B-cell epitopes, whereas AAY linkers were introduced between CTL epitopes to enhance proteasomal cleavage and improve MHC class I presentation. Similarly, GPGPG linkers were incorporated between HTL epitopes to facilitate MHC class II processing and promote efficient activation of CD4 + T-cell-mediated immune responses. To enhance immunogenicity, β-defensin-3 was positioned at the N-terminal region of the construct as an adjuvant. Additionally, a 6×His tag was appended at the C-terminal end to facilitate purification during recombinant expression and downstream processing. The final vaccine sequence was subsequently evaluated for antigenicity and allergenicity using VaxiJen v3.0 and AllerTOP v2.1 to assess its predicted immunostimulatory potential and safety profile^25^.

### Physicochemical properties analysis

The designed vaccine sequence was subjected to in silico evaluation of its physicochemical properties using the ExPASy ProtParam server (https://web.expasy.org/protparam/)^26,27^. This analysis was performed to determine key biochemical parameters, including molecular weight, theoretical isoelectric point (pI), instability index, aliphatic index, and grand average of hydropathicity (GRAVY), which collectively provide insights into the stability and overall physicochemical characteristics of the protein construct. Furthermore, the solubility of the construct in an *Escherichia coli* expression system was predicted using SoluProt v1.0 (https://loschmidt.chemi.muni.cz/soluprot)^28^.

### Structure modeling and validation

The tertiary structure of the multi-epitope vaccine construct was generated using AlphaFold3 (https://alphafoldserver.com/), which applies deep learning-based modeling to predict accurate three-dimensional conformations from amino acid sequences^29^. Following prediction, the initial model was subjected to structural optimization using GalaxyRefine (https://galaxy.seoklab.org/cgi-bin/submit.cgi?type=REFINE) to improve stereochemical quality and reduce structural irregularities such as steric clashes and unfavorable side-chain orientations^30^. The refined structure was then evaluated for stereochemical integrity using Ramachandran plot analysis through the PROCHECK module available within the UCLA-DOE LAB SAVES v6.1 server (http://services.mbi.ucla.edu/SAVES/)^31^. This assessment was used to examine residue conformations and the overall backbone geometry of the protein model. Additionally, structural reliability was further assessed using the Z-score obtained from ProSA-web (https://prosa.services.came.sbg.ac.at/prosa.php), which compares the predicted model with experimentally determined protein structures to evaluate overall model quality and identify potential structural errors^32^.

### Discontinuous B-cell epitope prediction

The three-dimensional structure of the vaccine construct was analyzed to identify conformational B-cell epitopes using ElliPro available through the IEDB platform (https://tools.iedb.org/ellipro/)^33^. This approach evaluates the spatial arrangement of amino acid residues on the protein surface and groups residues that are likely to form antibody-recognizable regions in the folded structure. The method relies on surface accessibility and residue protrusion indices to predict potential discontinuous epitope sites, which are important for understanding antigen–antibody recognition in a realistic structural context.

### Molecular docking and interactions analysis

Molecular docking studies were performed to evaluate the binding behavior of the designed vaccine construct with the innate immune receptors TLR3 and TLR4 using the ClusPro server (https://cluspro.bu.edu)^34^. TLR3 and TLR4 were selected as target receptors because of their established roles in antiviral innate immunity^35,36^. TLR3 recognizes viral double-stranded RNA (dsRNA)^37,38^, and Eyach virus belongs to the family *Spinareoviridae*, members of which possess segmented dsRNA genomes^1^. In contrast, TLR4 is a surface-expressed pattern recognition receptor involved in the activation of innate and adaptive immune responses^36,39^. Therefore, these receptors were selected to evaluate the immunostimulatory potential of the designed vaccine construct. The resulting docked complexes were further analyzed to investigate detailed molecular interactions using the PDBsum server (https://www.ebi.ac.uk/thornton-srv/databases/pdbsum/Generate.html)^40^. This analysis provided information on hydrogen bonds, salt bridges, and hydrophobic contacts, allowing a comprehensive evaluation of the stability and strength of the vaccine–receptor interactions.

### Molecular dynamics simulations

Molecular dynamics simulations were conducted to investigate the stability and dynamic behavior of the vaccine–receptor complexes under physiological conditions. The docked complexes formed between the vaccine construct and TLR3 or TLR4 were selected for simulation and analyzed using the Desmond module of the Schrödinger Suite^41^. Before initiating the simulation, the systems were prepared using the Maestro Protein Preparation Wizard to ensure structural correctness. This involved correcting bond orders, adding missing hydrogen atoms, optimizing hydrogen-bonding networks, and assigning appropriate protonation states to residues. The prepared complexes were then embedded in orthorhombic simulation boxes and solvated using the TIP3P water model to mimic a physiological aqueous environment^42,43^. To achieve system neutrality, counterions were introduced, followed by the addition of Na⁺ and Cl⁻ ions to simulate a physiological ionic strength of 0.15 M. The molecular dynamics simulations were performed under the OPLS-2005 force field using an NPT ensemble, maintaining a constant temperature of 300 K and pressure of 1 atm. Long-range electrostatic interactions were treated using the particle mesh Ewald (PME) method, while periodic boundary conditions were applied throughout the simulation. Each system was simulated for 100 ns to evaluate its dynamic behavior over time. Post-simulation trajectory analyses were performed to assess structural stability and flexibility. Root-mean-square deviation (RMSD) was used to evaluate overall conformational stability, whereas root-mean-square fluctuation (RMSF) provided residue-level flexibility information. The radius of gyration (Rg) was calculated to determine the compactness of the complexes during the simulation period. In addition, hydrogen-bond analysis was performed to examine the persistence of key intermolecular interactions. Finally, binding free energy estimation was carried out using the MM/GBSA approach to quantify the interaction strength and stability of the vaccine–receptor complexes.

### Immune simulations

The immune response elicited by the constructed vaccine was predicted using C-ImmSim (https://kraken.iac.rm.cnr.it/C-IMMSIM/index.php)^44^. The simulation was carried out over 1050 discrete time steps, corresponding to an overall period of 350 days, with each step representing 8 h of biological time. To maintain consistency in computational modeling, a seed value of 12345 and a simulation volume of 10 were assigned. A prime–boost vaccination regimen was implemented at time steps 1, 84, and 168. Each administration consisted of 1000 antigen units, while the adjuvant strength was fixed at 100. Lipopolysaccharide (LPS) was excluded from the simulation environment to ensure that the predicted immune response was solely attributed to the vaccine construct.

### Codon optimization and in silico cloning

To assess the feasibility of recombinant expression, the final multi-epitope vaccine protein sequence was first converted into a corresponding nucleotide sequence using the EMBOSS Backtranseq tool^45^. The generated DNA sequence was subsequently subjected to codon optimization based on the codon usage bias of *Escherichia coli* K12 through the ExpOptimizer server to enhance its predicted expression efficiency in the bacterial host. For cloning simulation, NdeI and XhoI restriction enzyme recognition sites were incorporated at the 5′ and 3′ termini of the optimized sequence, respectively. The modified gene sequence was then virtually inserted into the pET28a(+) expression vector using SnapGene software, allowing verification of correct insert orientation, maintenance of the open reading frame, and overall plasmid architecture prior to potential experimental validation^25^.

## Results

### Retrieval of protein

**Fig. 2:**
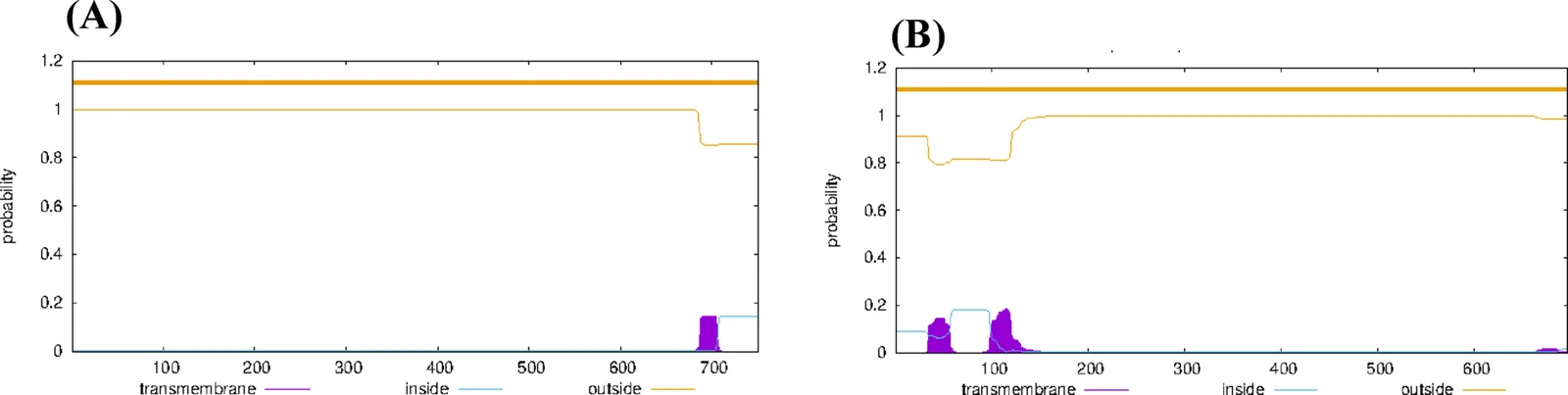
Transmembrane topology prediction of Eyach virus proteins using the TMHMM server. **(A)** VP5 and **(B)** VP7. The plots show the predicted probabilities of outside, inside, and transmembrane regions across the protein sequences.

The protein sequences of VP5 (NCBI ID: QYV43097.1) and VP7 (NCBI ID: QYV43099.1) of Eyach virus were retrieved from the NCBI database and subjected to preliminary evaluation of their functional characteristics, antigenicity, allergenicity, and transmembrane topology (Table 1). The antigenicity assessment indicated that VP5 possesses an antigenicity probability of 66%, whereas VP7 exhibited a higher probability of 100%, reflecting variation in their predicted immunogenic properties. Both proteins were predicted to be non-allergenic according to the allergenicity prediction model used. Transmembrane topology analysis using the TMHMM server revealed the absence of transmembrane helices in both VP5 and VP7 (Table 1). The graphical outputs further demonstrated that the amino acid residues of both proteins were predominantly distributed in the outside region, with negligible probabilities associated with transmembrane segments (Fig. 2). No distinct membrane-spanning domains were observed across the entire sequence length of either protein. Based on their biological and immunological characteristics, VP5 and VP7 were selected for subsequent vaccine design analyses. VP5 is an outer capsid protein involved in viral entry and host interaction, whereas VP7 is an outer capsid glycoprotein associated with viral infectivity and host–virus interactions. In addition to their important biological roles, both proteins were predicted to be antigenic and non-allergenic. Furthermore, transmembrane topology analysis demonstrated the absence of transmembrane helices, with the majority of residues localized in exposed regions. These characteristics suggest that VP5 and VP7 are accessible to host immune surveillance and represent suitable targets for epitope-based vaccine development.

**Table 1:** Selected Eyach virus proteins (VP5 and VP7) with their NCBI accession IDs, predicted antigenicity, allergenicity, and transmembrane topology characteristics.

Protein Name	NCBI Accession ID	Function	Antigenicity	Allergenicity	Subcellular Localization		
					Outside	Inside	TMhelix
VP5	QYV43097.1	Structural outer capsid protein involved in viral entry and host interaction	Probable immunogen with a probability of 66%	Non-Allergen	1–751	-	-
VP7	QYV43099.1	Structural outer capsid glycoprotein involved in host cell attachment	Probable immunogen with a probability of 100%	Non-Allergen	1–697	-	-

### Epitope prediction

Linear B-cell epitopes were predicted from the selected Eyach virus proteins VP5 and VP7, and the shortlisted epitopes were further evaluated for antigenicity, allergenicity, and toxicity (Table 2). From VP5, the epitope sequence ALMIHDPKTRQFKKLK was selected, whereas IVKLQNTNESNKKSLI was selected from VP7 based on the applied screening criteria. Antigenicity assessment indicated that both epitopes were probable immunogens, with the VP5-derived epitope showing an antigenicity probability of 66% and the VP7-derived epitope exhibiting a probability of 100%. In addition, allergenicity prediction classified both epitopes as non-allergenic, while toxicity analysis indicated that both peptides were non-toxic.

**Table 2:** Selected B cell-epitopes.

Protein Name	Epitopes	Antigenicity	Allergenicity	Toxicity
VP5	ALMIHDPKTRQFKKLK	Probable immunogen with a probability of 66%	Non-Allergen	Non-Toxic
VP7	IVKLQNTNESNKKSLI	Probable immunogen with a probability of 100%	Non-Allergen	Non-Toxic

CTL epitopes corresponding to MHC class I molecules were predicted from the selected VP5 and VP7 proteins and subsequently screened using the predefined selection criteria (Table 3). A total of six CTL epitopes were shortlisted based on their antigenicity, non-allergenicity, non-toxicity, percentile rank of less than 1, and interaction with multiple HLA class I alleles. All selected CTL epitopes were predicted to be probable immunogens with an antigenicity probability of 100%, while also being classified as non-allergenic and non-toxic. Furthermore, each selected epitope demonstrated the ability to bind multiple HLA alleles, indicating broad HLA class I allele coverage.

**Table 3:** Selected MHC class I epitopes.

Protein Name	Epitopes	Antigenicity	Allergenicity	Toxicity	Alleles
VP5	AIPKGVMAL	Probable immunogen with a probability of 100%	Non-Allergen	Non-Toxic	HLA-A*02:06, HLA-A*02:03 ,HLA-A*02:01, HLA-A*26:01, HLA-B*07:02
	FQDKVPKSL	Probable immunogen with a probability of 100%	Non-Allergen	Non-Toxic	HLA-A*02:06, HLA-A*02:01, HLA-B*08:01, HLA-A*02:03, HLA-B*40:01, HLA-B*15:01
	IHDPKTRQF	Probable immunogen with a probability of 100%	Non-Allergen	Non-Toxic	HLA-A*23:01, HLA-A*24:02, HLA-B*08:01, HLA-B*53:01, HLA-A*30:02, HLA-B*44:02, HLA-B*35:01, HLA-B*58:01
VP7	FTISFSHLI	Probable immunogen with a probability of 100%	Non-Allergen	Non-Toxic	HLA-A*68:02, HLA-A*02:06, HLA-A*26:01, HLA-B*51:01, HLA-A*32:01, HLA-A*02:01, HLA-B*58:01, HLA-A*02:03, HLA-B*57:01
	ISFSHLIAI	Probable immunogen with a probability of 100%	Non-Allergen	Non-Toxic	HLA-A*32:01, HLA-B*51:01, HLA-A*02:06, HLA-B*58:01, HLA-A*68:02, HLA-B*57:01, HLA-A*30:01
	PAVPSIQTY	Probable immunogen with a probability of 100%	Non-Allergen	Non-Toxic	HLA-B*35:01, HLA-B*53:01, HLA-A*26:01, HLA-A*30:02, HLA-B*58:01, HLA-B*15:01, HLA-A*01:01, HLA-B*57:01

HTL epitopes corresponding to MHC class II molecules were predicted and subsequently filtered based on antigenicity, allergenicity, toxicity, percentile rank of less than 10, and interaction with multiple HLA class II alleles (Table 4). The selected epitopes from both VP5 and VP7 proteins satisfied the predefined selection criteria and were therefore retained for further analysis. Antigenicity assessment revealed variability among the selected epitopes, with VP5-derived epitopes showing an antigenicity probability of 66%, whereas VP7-derived epitopes exhibited a probability of 100%. All selected HTL epitopes were predicted to be non-allergenic and non-toxic. In addition, each epitope demonstrated the ability to interact with multiple HLA class II alleles, indicating broad HLA class II allele coverage.

**Table 4:** Selected MHC class II epitopes.

Protein Name	Epitopes	Antigenicity	Allergenicity	Toxicity	Alleles
VP5	NFGRFVIAMDPRMNL	Probable immunogen with a probability of 66%	Non-Allergen	Non-Toxic	HLA-DRB1*03:01, HLA-DRB1*04:01, HLA-DRB1*04:05, HLA-DRB1*01:01, HLA-DRB1*09:01, HLA-DRB1*07:01
	RNGYRVSRAAIPKGV	Probable immunogen with a probability of 66%	Non-Allergen	Non-Toxic	HLA-DRB1*09:01, HLA-DRB1*07:01, HLA-DRB1*08:02, HLA-DRB1*04:01, HLA-DRB1*01:01, HLA-DRB1*11:01, HLA-DRB1*04:05, HLA-DRB1*13:02
VP7	QRGTVSLLEAEFAAL	Probable immunogen with a probability of 100%	Non-Allergen	Non-Toxic	HLA-DRB1*01:01, HLA-DRB1*12:01
	SLLEAEFAALKKTHQ	Probable immunogen with a probability of 100%	Non-Allergen	Non-Toxic	HLA-DRB1*11:01, HLA-DRB1*08:02, HLA-DRB1*13:02

### Epitope conservancy analysis

The conservancy of the selected B-cell, CTL, and HTL epitopes was evaluated across the available Eyach virus VP5 and VP7 protein sequences retrieved from the NCBI database (Tables 5 and Table 6). For VP5, most of the selected epitopes exhibited complete conservation (100%) across all analyzed sequences (Table 5). The B-cell epitope ALMIHDPKTRQFKKLK, CTL epitopes AIPKGVMAL and IHDPKTRQF, and HTL epitope RNGYRVSRAAIPKGV were fully conserved in all three VP5 sequences. Minor sequence variation was observed for the CTL epitope FQDKVPKSL and the HTL epitope NFGRFVIAMDPRMNL in the sequence NP_620284.1, resulting in conservancy values of 88.89% and 93.33%, respectively. Nevertheless, these epitopes remained highly conserved among the analyzed VP5 sequences.

Similarly, all selected epitopes derived from VP7 demonstrated complete conservation (100%) across the two available VP7 sequences analyzed in this study (Table 6). The B-cell epitope IVKLQNTNESNKKSLI, CTL epitopes FTISFSHLI, ISFSHLIAI, and PAVPSIQTY, as well as HTL epitopes QRGTVSLLEAEFAAL and SLLEAEFAALKKTHQ, showed identical amino acid sequences in both VP7 isolates. Overall, the high degree of epitope conservancy observed for both VP5 and VP7 suggests that the selected epitopes are largely preserved among the currently available Eyach virus sequences.

**Table 5:** Conservancy of selected B-cell, CTL, and HTL epitopes from VP5.

Epitopes	B-Cell epitopes	MHC-I epitopes			MHC-II epitopes	
	ALMIHDPKTRQFKKLK	AIPKGVMAL	FQDKVPKSL	IHDPKTRQF	NFGRFVIAMDPRMNL	RNGYRVSRAAIPKGV
**Accession Id**						
QYV43097.1	100%	100%	100%	100%	100%	100%
AND77152.1	100%	100%	100%	100%	100%	100%
NP_620284.1	100%	100%	88.89%	100%	93.33%	100%

**Table 6:** Conservancy of selected B-cell, CTL, and HTL epitopes from VP7.

Epitopes	B-Cell epitopes	MHC-I epitopes			MHC-II epitopes	
	IVKLQNTNESNKKSLI	FTISFSHLI	ISFSHLIAI	PAVPSIQTY	QRGTVSLLEAEFAAL	SLLEAEFAALKKTHQ
**Accession Id**						
QYV43099.1	100%	100%	100%	100%	100%	100%
NP_620286.1	100%	100%	100%	100%	100%	100%

### Population coverage analysis

**Fig. 3:**
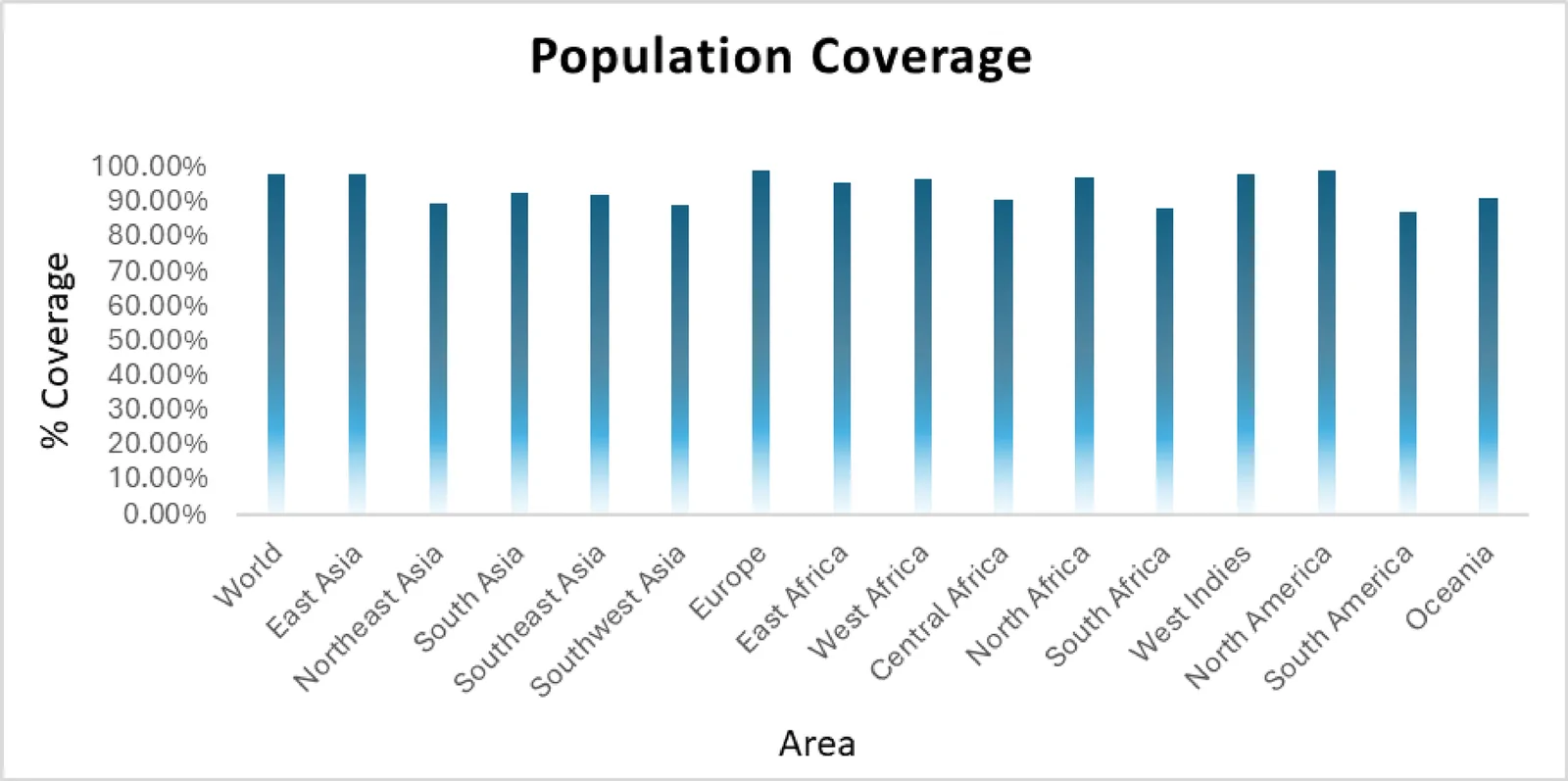
Predicted population coverage of the selected T-cell epitopes in different geographic regions.

The population coverage of the selected T-cell epitopes was evaluated across different geographic regions using the IEDB population coverage tool, and the results are presented in Fig. 3. The analysis demonstrated broad population coverage worldwide, with values ranging from 86.95% to 99.19%. The overall global population coverage was estimated at 97.94%. Among the analyzed regions, Europe exhibited the highest coverage (99.19%), followed by North America (98.86%), East Asia (98.27%), and the West Indies (98.25%). High coverage was also observed in North Africa (96.97%), West Africa (96.44%), East Africa (95.57%), Central Africa (90.79%), and South Africa (88.28%). In comparison, Southeast Asia, South Asia, Northeast Asia, Southwest Asia, South America, and Oceania showed coverage values of 91.93%, 92.47%, 89.61%, 89.16%, 86.95%, and 91.12%, respectively. Overall, these findings indicate that the selected epitopes provide broad predicted population coverage through their association with diverse HLA alleles across different geographic regions.

### Vaccine construct assemblage

**Fig. 4:**
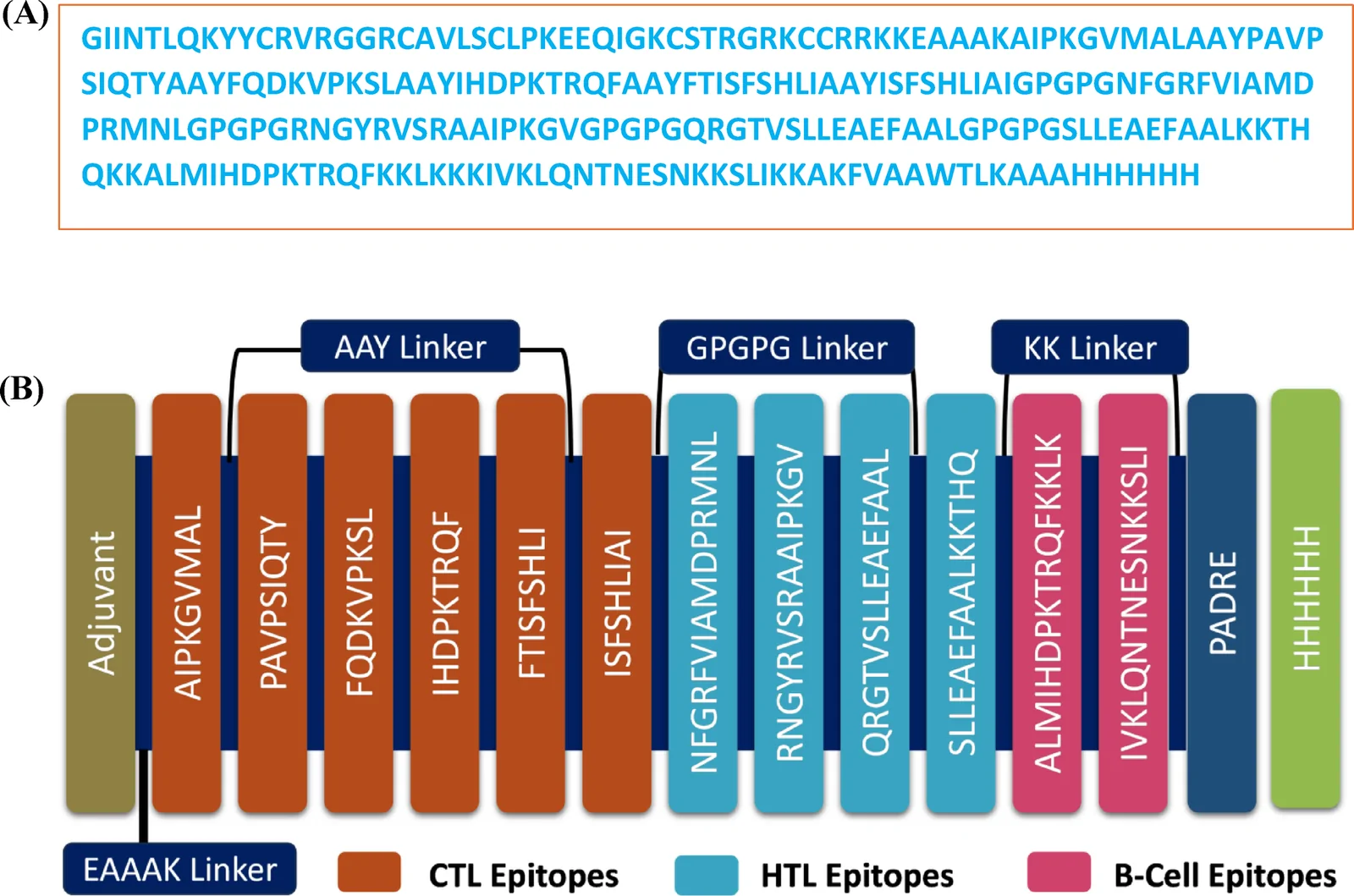
**(A)** Linear sequence of the designed vaccine construct. **(B)** Schematic representation of the designed multi-epitope vaccine construct illustrating the linear arrangement of the β-defensin-3 adjuvant, EAAAK linker, KK-linked B-cell epitopes, AAY-linked CTL epitopes, GPGPG-linked HTL epitopes, and the C-terminal 6×His tag.

The multi-epitope vaccine construct was designed by integrating the selected immunogenic components into a single continuous polypeptide sequence. An immunostimulatory adjuvant, β-defensin-3, was positioned at the N-terminal end and linked to the epitope regions using an EAAAK linker to ensure appropriate structural separation and stability (Fig. 4). The construct includes six CTL epitopes, which were sequentially joined using AAY linkers to facilitate proper processing and presentation through MHC class I pathways. Four HTL epitopes were incorporated and connected using GPGPG linkers to support MHC class II-mediated immune responses. Two B-cell epitopes were also included in the construct and linked using KK linkers. To support purification during recombinant expression, a 6×His tag was added at the C-terminal end of the sequence. A PADRE sequence was incorporated into the construct prior to the C-terminal 6×His tag. The final construct comprised 256 amino acids. Computational analysis predicted the construct to be antigenic, with an antigenicity probability of 100%, while also being classified as non-allergenic based on VaxiJen v3.0 and AllerTOP v2.1 predictions.

### Physicochemical properties analysis

The physicochemical characteristics of the designed vaccine construct were evaluated using the ProtParam and SoluProt servers, and the results are summarized in Table 7. The construct consists of 256 amino acids with a calculated molecular weight of 27,965.86 Da. The theoretical isoelectric point (pI) was determined to be 10.28, indicating the basic nature of the protein. The instability index was calculated as 18.77, which is below the threshold value of 40, suggesting that the construct is stable according to the prediction model. The aliphatic index was found to be 79.06, reflecting the relative abundance of aliphatic side chains within the protein. The grand average of hydropathicity (GRAVY) score was − 0.292, indicating a predominantly hydrophilic character. Furthermore, the predicted solubility score of 0.832 suggests that the construct is likely to be soluble upon heterologous expression.

**Table 7:** Predicted physicochemical properties of the designed multi-epitope vaccine construct.

Parameters	Results
Number of amino acids	256
Theoretical pI	10.28
Molecular weight	27965.86
Instability index	18.77
Aliphatic index	79.06
Grand average of hydropathicity	−0.292
Solubility	0.832

### Structure modeling and validation

**Fig. 5:**
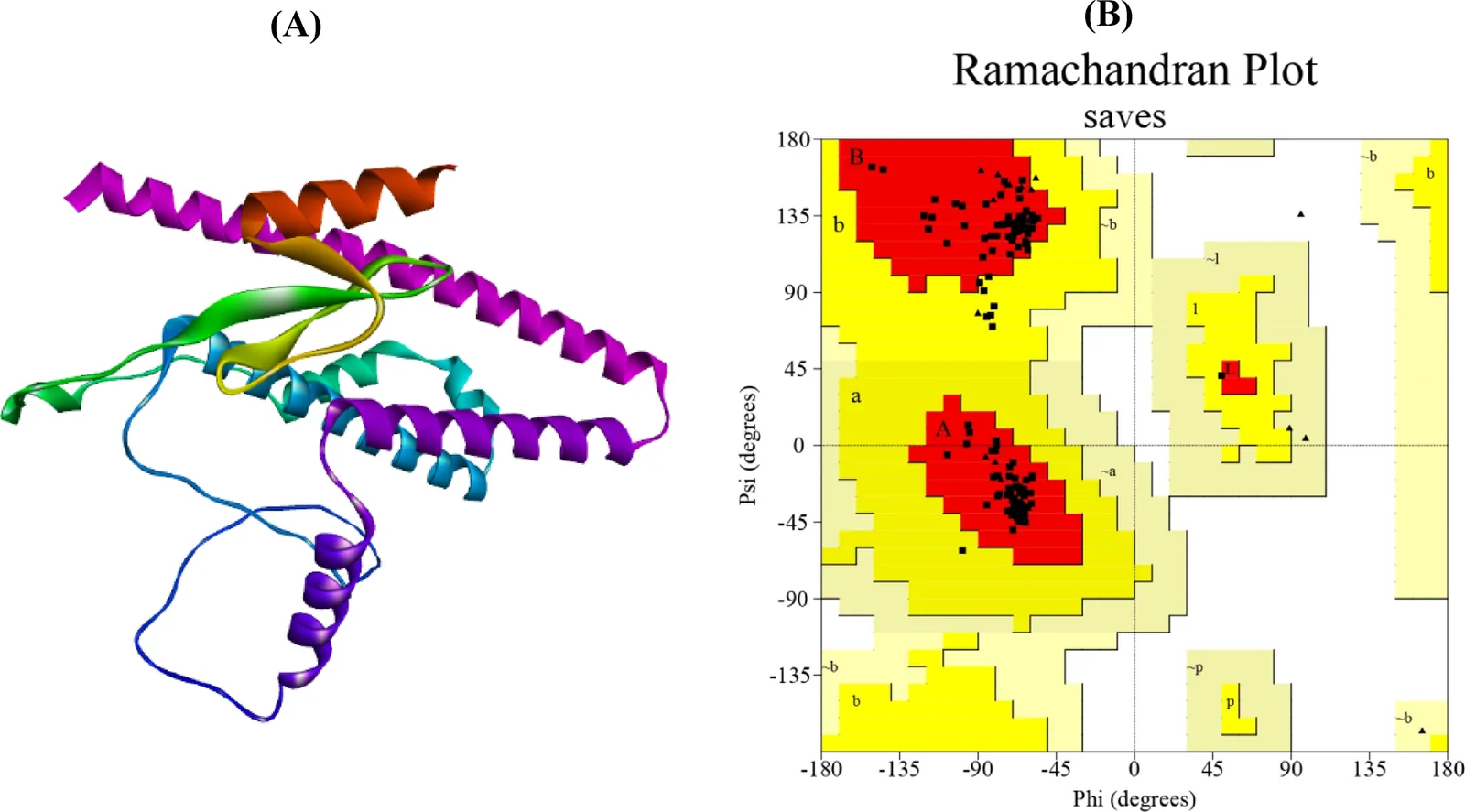
**(A)** Predicted three-dimensional structure of the vaccine construct modeled using AlphaFold3. **(B)** Ramachandran plot showing the distribution of backbone dihedral angles for stereochemical evaluation of the refined model.

The three-dimensional structure of the vaccine construct was predicted using AlphaFold3 and subsequently refined to improve its structural quality (Fig. 5A). The quality of the model was evaluated through Ramachandran plot analysis and ProSA Z-score assessment to examine residue geometry and overall model reliability. Before refinement, the Ramachandran plot indicated that 80.6% of residues were located in favored regions, 19.0% in allowed regions, 0.5% in generously allowed regions, and no residues were present in disallowed regions. Following refinement, the distribution improved, with 96.3% of residues located in favored regions and 3.7% in allowed regions, while residues in generously allowed and disallowed regions were absent (Fig. 5B). The ProSA analysis showed a Z-score of − 3.00 for the initial model, which improved slightly to − 3.17 after refinement. According to the ProSA-web server, Z-scores falling within the range typically observed for experimentally determined native protein structures of comparable size are indicative of acceptable overall model quality. The refined vaccine construct exhibited a Z-score of − 3.17, which falls within the range of native protein structures and therefore supports the reliability of the predicted model. Furthermore, the absence of major deviations in the ProSA analysis suggests that the refined structure does not contain significant structural abnormalities, supporting its suitability for subsequent docking and molecular dynamics simulation analyses.

### Discontinuous B-cell epitope prediction

**Fig. 6:**
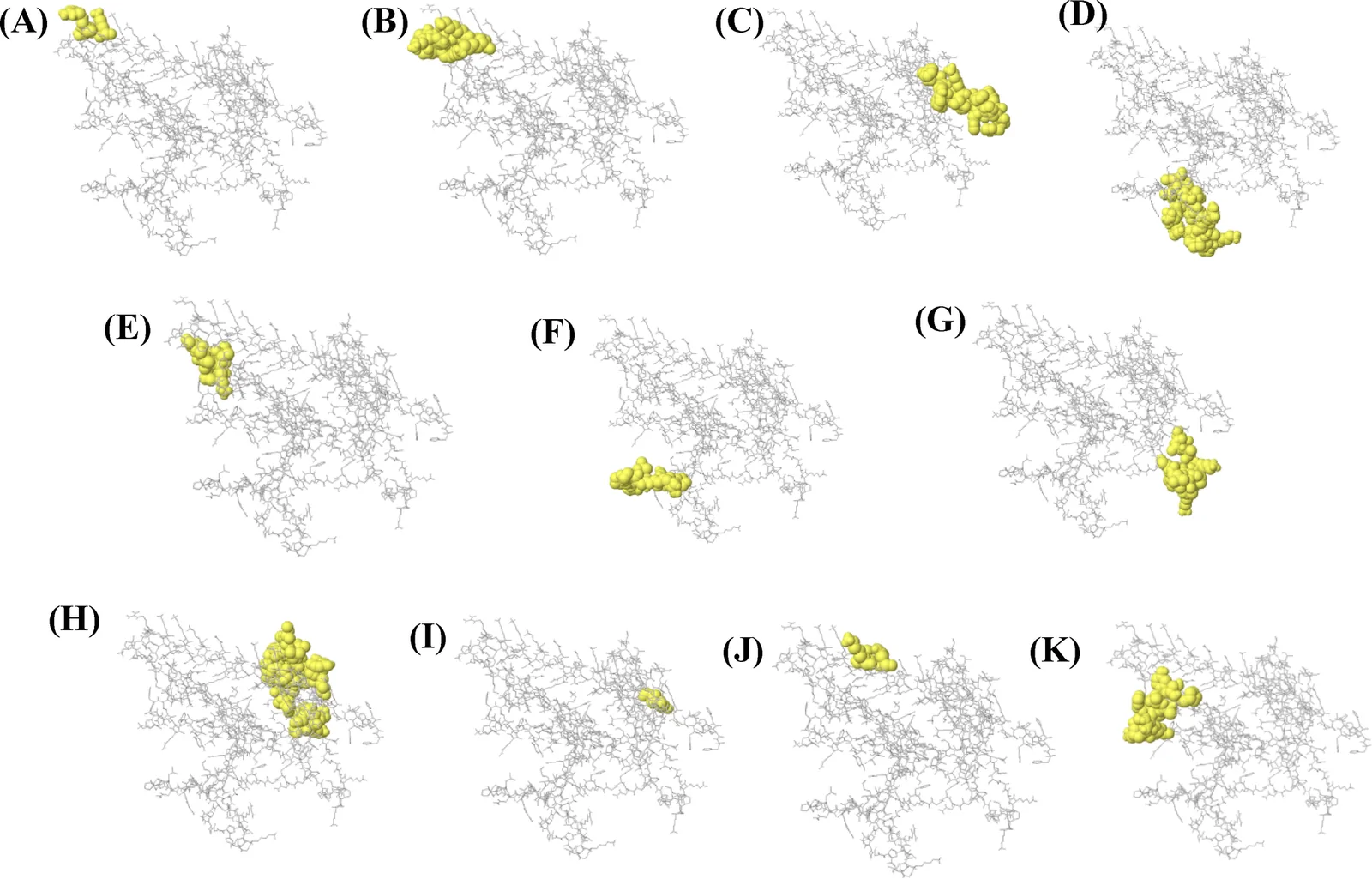
Three-dimensional visualization of ElliPro-predicted discontinuous B-cell epitopes mapped onto the multi-epitope vaccine construct. **(A)** Epitope (1) **(B)** Epitope (2) **(C)** Epitope (3) **(D)** Epitope (4) **(E)** Epitope (5) **(F)** Epitope (6) **(G)** Epitope (7) **(H)** Epitope (8) **(I)** Epitope (9) **(J)** Epitope (10) **(K)** Epitope 11.

Discontinuous B-cell epitopes of the vaccine construct were predicted using the ElliPro tool based on the refined three-dimensional structure of the model. A total of 11 conformational B-cell epitopes were identified, and their spatial distribution on the protein surface is illustrated in Fig. 6. The predicted epitopes varied in size, containing between 3 and 25 residues, as summarized in Table 8. ElliPro scores ranged from 0.581 to 0.948, indicating differences in the predicted protrusion and surface accessibility of the epitopic regions. The highest score (0.948) was observed for epitope 1, whereas the remaining epitopes exhibited scores between 0.581 and 0.849. Larger conformational epitopes were also identified, including epitope 8 comprising 25 residues and epitope 4 comprising 22 residues. Overall, the predicted discontinuous epitopes were distributed across multiple surface-exposed regions of the vaccine construct, suggesting the presence of several potential antibody-accessible sites.

**Table 8:** Predicted conformational B-cell epitopes of the vaccine construct.

Sr. No.	Residues	Number of residues	Score
1	V: T210, V: R211, V: K214	3	0.948
2	V: D207, V: P208, V: K209, V: Q212, V: F213, V: K215, V: L216, V: K217, V: K219	9	0.849
3	V: F240, V: A243, V: W244, V: T245, V: L246, V: K247, V: A248, V: A249, V: A250, V: H251, V: H252, V: H253, V: H254, V: H255, V: H256	15	0.813
4	V: V150, V: S151, V: R152, V: A153, V: A154, V: I155, V: P156, V: K157, V: G158, V: V159, V: G160, V: P161, V: G162, V: P163, V: G164, V: Q165, V: R166, V: G167, V: T168, V: V169, V: S170, V: L171	22	0.794
5	V: K201, V: A202, V: L203, V: M204, V: I205, V: H206	6	0.775
6	V: N138, V: L139, V: G140, V: P141, V: G142, V: P143, V: G144, V: R145, V: N146, V: G147, V: Y148	11	0.77
7	V: E46, V: A47, V: A48, V: A49, V: K50, V: A51, V: I52, V: P53, V: K54, V: G55, V: V56, V: M57, V: A58, V: L59, V: A60	15	0.754
8	V: G1, V: I2, V: N4, V: T5, V: L6, V: K8, V: Y9, V: R12, V: V13, V: C18, V: A19, V: V20, V: L21, V: S22, V: C23, V: L24, V: P25, V: K26, V: E27, V: C33, V: S34, V: R36, V: G37, V: R38, V: R43	25	0.628
9	V: G15, V: G16, V: R17	3	0.598
10	V: V221, V: K222, V: N225	3	0.586
11	V: P80, V: S82, V: A84, V: A85, V: Y86, V: I87, V: H88, V: D89, V: P90, V: K91, V: R93	11	0.581

#### Molecular docking and interaction analysis

Molecular docking was performed to evaluate the interaction between the vaccine construct and the immune receptors TLR3 and TLR4. For both complexes, cluster 0 was identified as the most representative cluster, each comprising 87 members, indicating the presence of multiple similar docking conformations. The vaccine construct–TLR3 complex exhibited the lowest binding energy of −1380.7, whereas the vaccine construct–TLR4 complex showed the lowest binding energy of −1073.9. In protein–protein docking, more negative docking energies generally indicate more favorable predicted interactions. However, because docking energies are influenced by factors such as protein size, structural conformation, and interface characteristics, these values are primarily used for comparative assessment rather than as absolute measures of binding affinity. Under identical docking conditions, the lower energy observed for the vaccine–TLR3 complex suggests a relatively more favorable predicted interaction than that observed for the vaccine–TLR4 complex. These values reflect differences in the predicted interaction energies between the vaccine construct and the respective receptors.

**Fig. 7:**
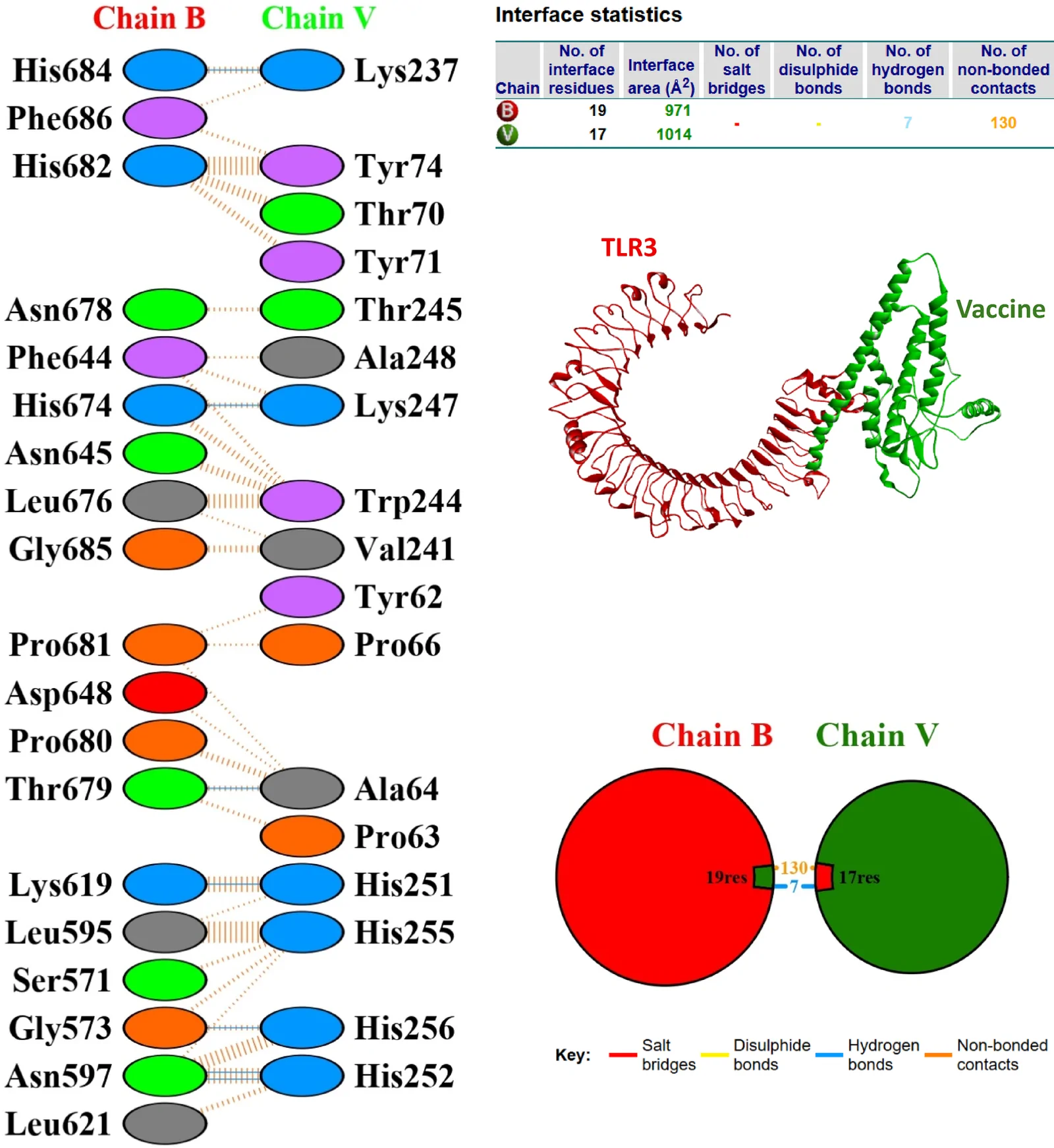
Interaction map of the vaccine–TLR3 complex showing residue-level contacts between receptor (chain B) and vaccine construct (chain V).

The interaction characteristics of the vaccine–TLR3 complex were analyzed to examine residue-level contacts at the binding interface. The interface involved 19 residues from the receptor (chain B) and 17 residues from the vaccine construct (chain V), with interface areas of 971 Å² and 1014 Å², respectively. A total of 7 hydrogen bonds, represented by blue lines in Fig. 7, were observed between the interacting residues of the two chains. These hydrogen bonds were formed between receptor residues His684, His682, Asn678, His674, Lys619, Gly573, and Asn597 and vaccine residues Lys237, Tyr74, Thr245, Lys247, His251, His256, and His252. In addition to hydrogen bonding, 130 non-bonded contacts were identified between the interacting residues. These contacts involved multiple amino acids from both chains, including residues such as Phe686, Asn645, Leu676, Pro681, Asp648, Thr679, and Ser571 from the receptor (chain B), interacting with residues such as Tyr71, Ala248, Trp244, Val241, Pro66, Ala64, and Pro63 from the vaccine construct (chain V). No salt bridges or disulfide bonds were detected within the analyzed complex. The interaction map further showed that contacts were distributed across multiple regions of both the receptor and the vaccine construct, reflecting the interaction pattern observed at the binding interface.

**Fig. 8:**
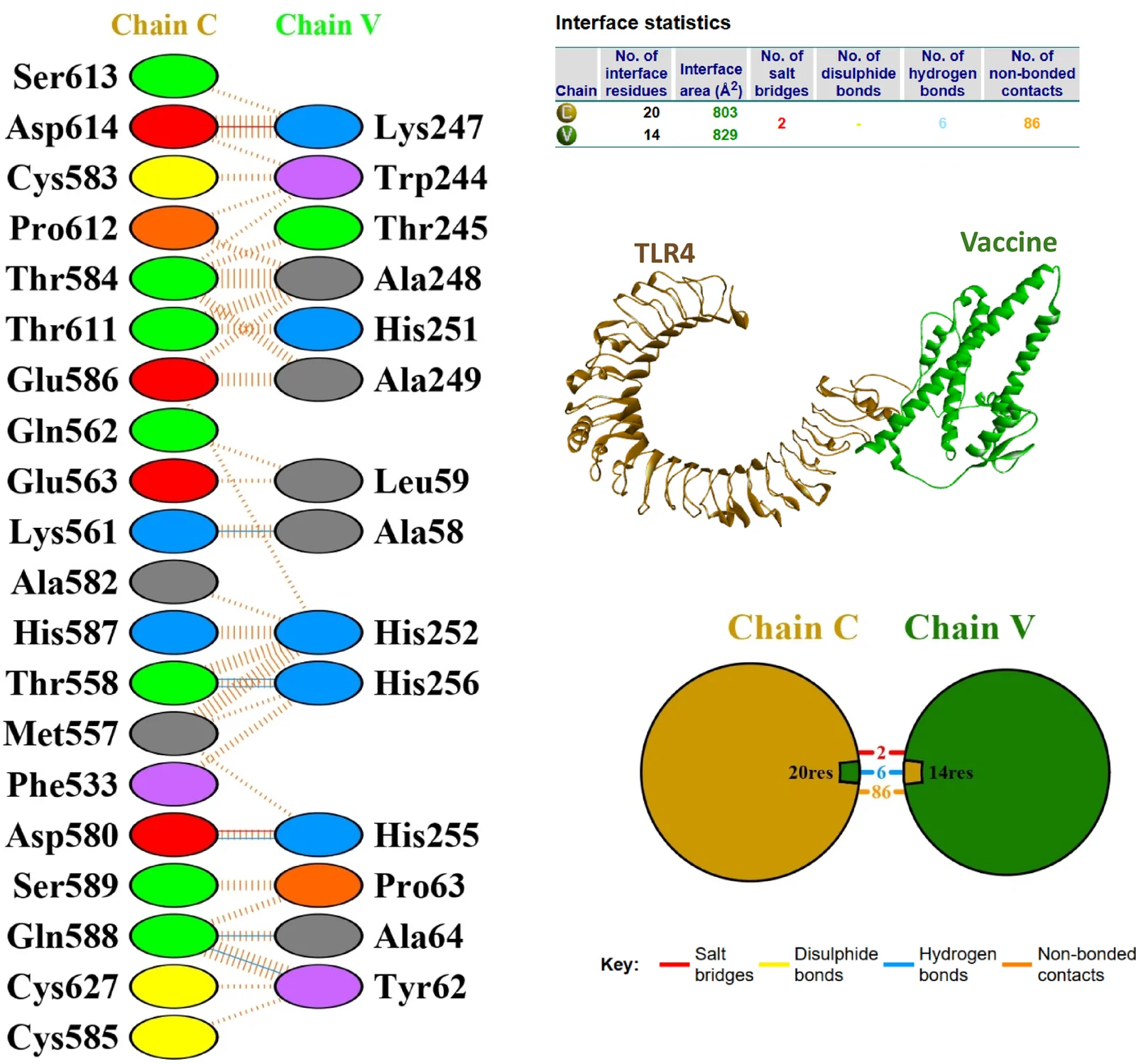
Interaction map of the vaccine–TLR4 complex showing residue-level contacts between receptor (chain C) and vaccine construct (chain V).

The interaction profile of the vaccine–TLR4 complex was analyzed to examine residue-level contacts at the binding interface. The interface involved 20 residues from the receptor (chain C) and 14 residues from the vaccine construct (chain V), with interface areas of 803 Å² and 829 Å², respectively. A total of 6 hydrogen bonds, represented by blue lines in Fig. 8, were observed between interacting residues of the two chains. These hydrogen bonds were formed between receptor residues Asp614, Lys561, His587, Thr558, and Asp580 and vaccine residues Lys247, Ala58, His252, His256, and His255. In addition, 2 salt bridges were identified at the interface, indicating ionic interactions between specific residue pairs. A total of 86 non-bonded contacts were also observed, involving multiple residues from both the receptor and the vaccine construct. These interactions included receptor residues such as Ser613, Cys583, Pro612, Thr584, Glu586, Gln562, and Phe533 interacting with vaccine residues such as Trp244, Thr245, Ala248, Ala249, Leu59, and Tyr62. No disulfide bonds were detected in the analyzed complex. The interaction map further illustrated that contacts were distributed across several regions of both the receptor and the vaccine construct, reflecting the interaction pattern observed at the binding interface (Fig. 8).

#### Molecular dynamics simulations

**Fig. 9:**
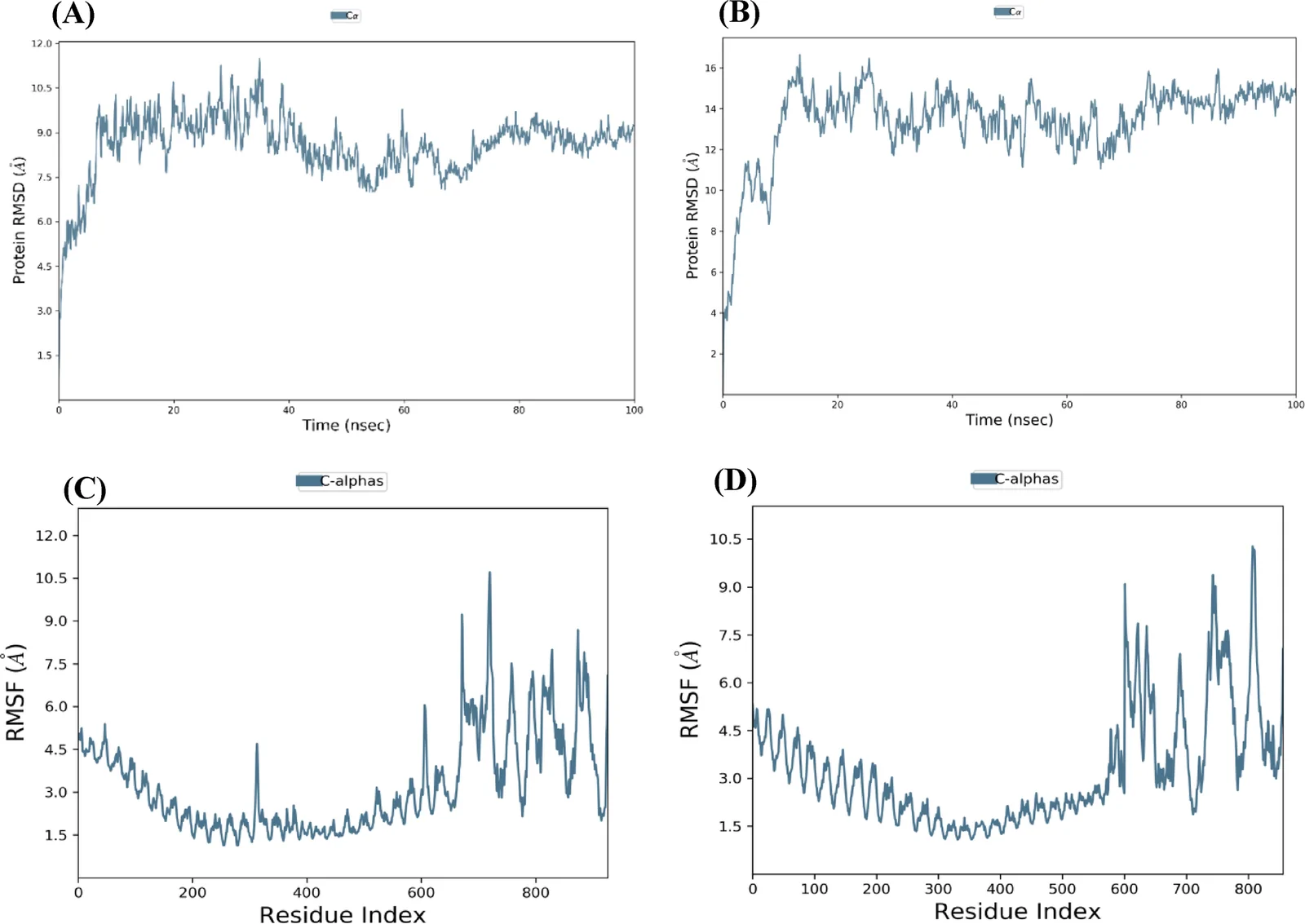
RMSD and RMSF analyses of vaccine–TLR complexes obtained from molecular dynamics simulations. **(A)** RMSD profile of the vaccine–TLR3 complex. **(B)** RMSD profile of the vaccine–TLR4 complex. **(C)** RMSF profile of the vaccine–TLR3 complex. **(D)** RMSF profile of the vaccine–TLR4 complex.

The dynamic behavior of the vaccine–receptor complexes was evaluated over a 100 ns simulation period. The RMSD profile of the vaccine–TLR3 complex (Fig. 9A) showed an initial increase from approximately 4.0 Å to 8.5–9.5 Å within the first 10 ns, indicating an early conformational adjustment. After this phase, the trajectory fluctuated within a narrower range of 8.0–10.5 Å for the remainder of the simulation. A slight decrease was observed between 40 and 70 ns, where RMSD values approached 7.5 Å, followed by a return toward approximately 9.0 Å by the end of the simulation. In contrast, the vaccine–TLR4 complex (Fig. 9B) exhibited a more pronounced initial increase, rising from approximately 3.5 Å to 14–16 Å within the first 10–15 ns. Following this transition, RMSD values fluctuated within a broader range of 13.0–15.5 Å throughout the simulation. A gradual increase was observed after 70 ns, with values approaching 15 Å toward the end of the trajectory. Comparatively, the vaccine–TLR3 complex displayed lower RMSD values and a narrower fluctuation range than the vaccine–TLR4 complex, indicating comparatively reduced structural deviation during the simulation period. The elevated RMSD values observed, particularly in the vaccine–TLR4 complex, likely reflect conformational adaptation and structural rearrangement of the vaccine construct during receptor engagement rather than complete destabilization of the complex. This behavior may be attributed, in part, to the presence of flexible linker sequences connecting the individual epitopes within the multi-epitope vaccine construct, which can contribute to increased structural mobility during the simulation while maintaining receptor–vaccine interactions.

The RMSF profiles provided residue-level insight into flexibility patterns within the complexes. For the vaccine–TLR3 complex (Fig. 9C), residues corresponding to TLR3 (residues 1–671) exhibited fluctuations primarily within 1.5–4.5 Å, with lower values of approximately 1.5–2.0 Å observed between residues 250 and 400. The vaccine construct region (residues 672–927) showed comparatively higher fluctuations, ranging from 3.0 to 10.5 Å, with prominent peaks observed around residues 700–780. The major fluctuating residues within the vaccine construct included Gly1–Ile2, Ala47–Ala51, His88–Asp89, Pro123–Phe126, Pro143–Gly144, Lys157–Gly160, Leu203–Asp207, and Lys214–Lys222. In contrast, only limited fluctuations were observed in the TLR3 receptor, primarily involving Ile338, Ser339, and Pro632. Comparison with the docking interface demonstrated that the majority of these fluctuating residues were located outside the TLR3–vaccine binding interface, indicating that the observed flexibility mainly reflects conformational adaptation of exposed and linker-associated regions of the vaccine construct rather than instability of the receptor–vaccine complex.

Similarly, the vaccine–TLR4 complex (Fig. 9D) showed lower fluctuations within the receptor region (residues 1–601), generally ranging from 1.2 to 4.5 Å, with minimum values of approximately 1.2–1.8 Å observed between residues 250 and 350. The vaccine region (residues 602–857) exhibited higher variability, with RMSF values ranging between 2.5 and 10.5 Å, including noticeable peaks around residues 650–780. The principal fluctuating residues within the vaccine construct were Gly1–Thr5, Val20–Ser22, Thr35–Arg38, Arg136, Gly142–Arg149, and Ile205–Arg211. Comparison with the docking interface revealed that these residues were predominantly located outside the TLR4–vaccine binding interface, suggesting that the observed fluctuations represent localized conformational flexibility rather than disruption of receptor binding.

When compared, both systems showed a similar pattern in which the receptor regions exhibited lower fluctuations relative to the vaccine construct. However, the vaccine–TLR3 complex demonstrated slightly more uniform fluctuation patterns, whereas the vaccine–TLR4 complex exhibited more pronounced peaks in the vaccine region, indicating relatively higher localized flexibility.

**Fig. 10:**
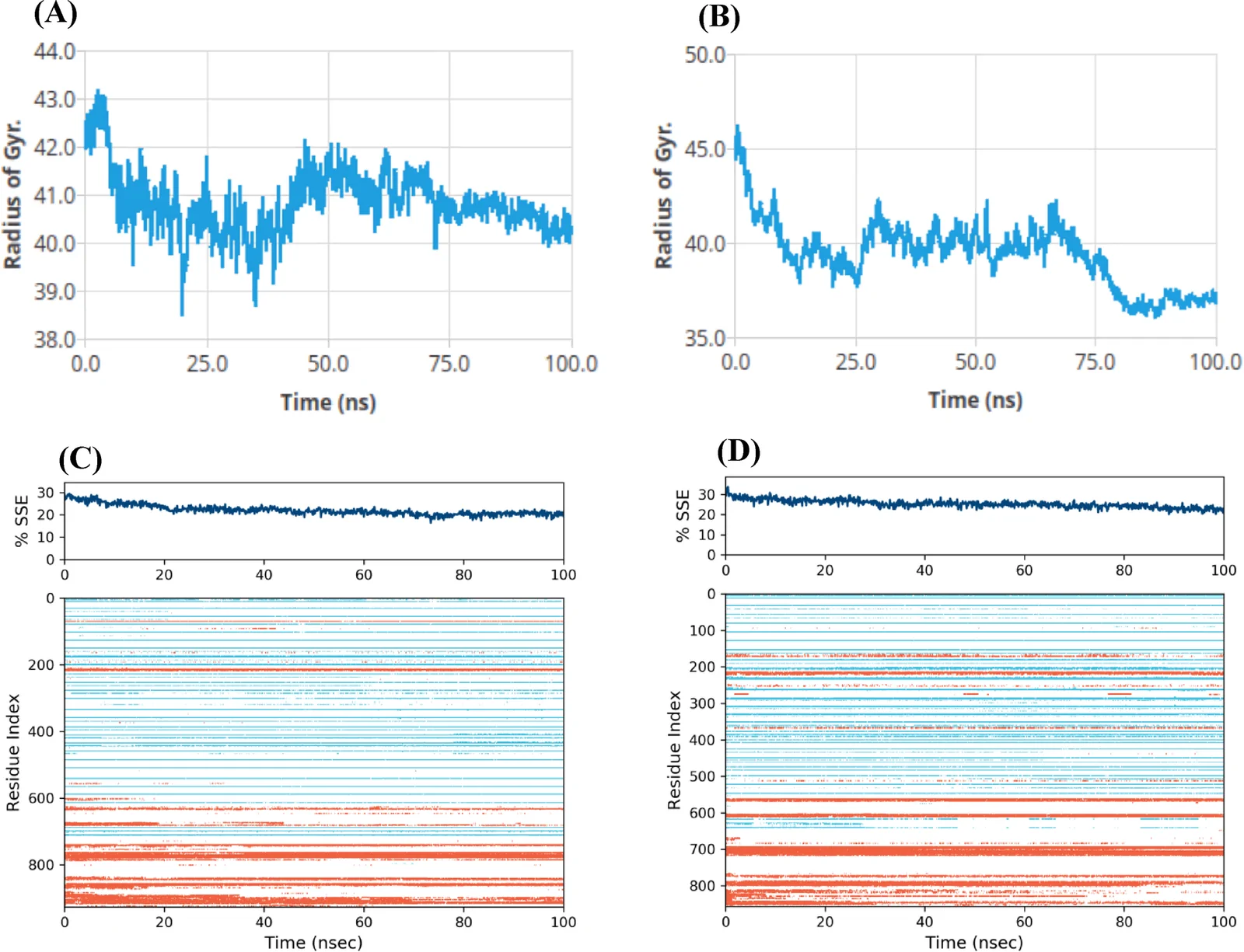
Radius of gyration (Rg) and secondary structure analysis of vaccine–TLR complexes. **(A)** Radius of gyration profile of the vaccine–TLR3 complex over 100 ns. **(B)** Radius of gyration profile of the vaccine–TLR4 complex over 100 ns. **(C)** Time-dependent evolution of secondary structure elements (SSE) for the vaccine–TLR3 complex. **(D)** Time-dependent evolution of secondary structure elements (SSE) for the vaccine–TLR4 complex.

The vaccine–TLR3 complex (Fig. 10A) exhibited Rg values ranging between 39.0 and 43.0 Å throughout the simulation. An initial decrease from approximately 42.5 Å to 40.5 Å was observed within the first 10–15 ns, followed by fluctuations within the range of 40.0–42.0 Å for the remainder of the trajectory. Minor increases were observed around 45–60 ns, where values approached approximately 42.0 Å, before returning closer to 40.5 Å toward the end of the simulation. In comparison, the vaccine–TLR4 complex (Fig. 10B) showed a broader variation in Rg values, ranging from 36.5 to 46.0 Å. A noticeable decrease occurred during the initial phase, where the Rg declined from approximately 45.5 Å to 40.0 Å within the first 15–20 ns. Following this phase, the values fluctuated between 38.5 and 41.5 Å, followed by a gradual decrease after 70 ns, reaching approximately 36.5–37.5 Å toward the end of the simulation. Comparatively, the vaccine–TLR3 complex maintained a narrower Rg range, whereas the vaccine–TLR4 complex exhibited greater variation and a gradual reduction in Rg values over time, indicating comparatively higher structural compaction during the later stages of the simulation.

For the vaccine–TLR3 complex (Fig. 10C), the total secondary structure element (SSE) content remained relatively consistent, fluctuating between approximately 22% and 27% throughout the simulation. The distribution pattern indicated the presence of persistent secondary structural elements interspersed with regions exhibiting structural transitions over time. The receptor region (lower residue indices) displayed relatively uniform patterns, whereas the vaccine region (higher residue indices) showed comparatively greater variation in structural elements. Similarly, the vaccine–TLR4 complex (Fig. 10D) exhibited SSE content fluctuating within a slightly broader range of approximately 23%–29%. The distribution pattern indicated the presence of persistent secondary structural elements throughout the simulation, with localized variations observed across specific residue segments. As observed for the vaccine–TLR3 complex, the vaccine-associated regions displayed comparatively greater variability than the receptor regions. When compared, both systems exhibited similar overall SSE percentages; however, the vaccine–TLR4 complex showed slightly greater variation in SSE content, whereas the vaccine–TLR3 complex maintained a more uniform distribution throughout the simulation period.

**Fig. 11:**
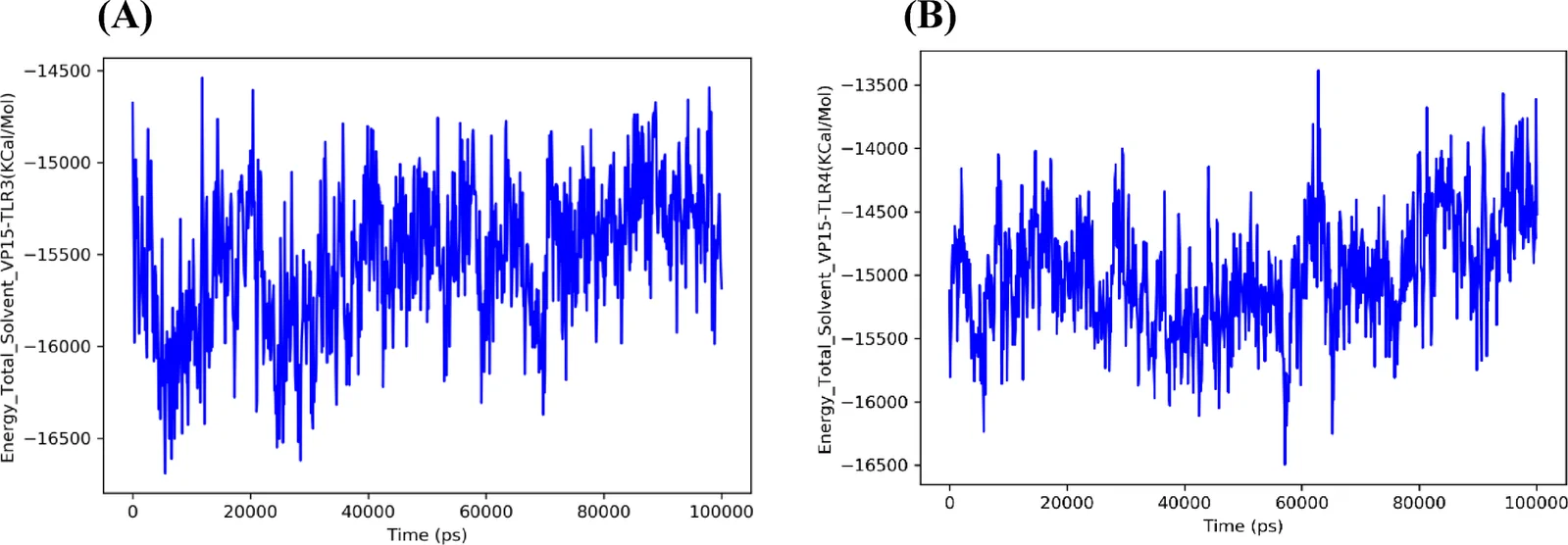
Total energy profiles of vaccine–TLR complexes during molecular dynamics simulations. **(A)** Total energy profile of the vaccine–TLR3 complex. **(B)** Total energy profile of the vaccine–TLR4 complex.

The total energy profiles of the vaccine–receptor complexes over the simulation period are shown in Fig. 11. The vaccine–TLR3 complex (Fig. 11A) exhibited total energy values fluctuating within a range of approximately −16,700 to −14,800 kcal/mol throughout the simulation. During the initial phase (0–10 ns), the system showed a noticeable decrease in energy, reaching values near −16,600 kcal/mol, followed by fluctuations around − 16,000 to − 15,200 kcal/mol for the remainder of the trajectory. Although short-term variations were evident, the energy values remained within a defined range over time. In comparison, the vaccine–TLR4 complex (Fig. 11B) showed total energy values ranging between approximately −16,500 and −13,500 kcal/mol. The trajectory displayed continuous fluctuations, with energy values most frequently observed around −15,500 to −14,500 kcal/mol. A few transient deviations were observed, including a brief decrease near −16,500 kcal/mol during the simulation and occasional increases approaching − 13,800 kcal/mol. When compared, the vaccine–TLR3 complex exhibited more negative total energy values overall, whereas the vaccine–TLR4 complex showed a comparatively wider energy distribution and slightly higher energy range. Both systems demonstrated continuous fluctuations without abrupt, sustained shifts in energy levels throughout the simulation period.

The MM-GBSA analysis results for the vaccine–TLR3 and vaccine–TLR4 complexes are presented in Table 9. The calculated binding free energy (ΔG_bind) for the vaccine–TLR3 complex was −203.29 kcal/mol, whereas the vaccine–TLR4 complex exhibited a value of −55.28 kcal/mol. According to the MM-GBSA calculations, the more negative ΔG_bind value obtained for the vaccine–TLR3 complex indicates comparatively more favorable predicted binding energetics than those observed for the vaccine–TLR4 complex. Analysis of the individual energy components showed that Coulombic interactions contributed substantially to binding in both systems, with values of −212.15 kcal/mol for TLR3 and −92.74 kcal/mol for TLR4. The magnitude of this contribution was higher in the TLR3 complex, indicating a comparatively greater electrostatic contribution within this system. The van der Waals (vdW) interactions also differed between the two complexes, with values of −166.96 kcal/mol for TLR3 and −43.65 kcal/mol for TLR4. Similarly, lipophilic interactions contributed −89.99 kcal/mol and −6.46 kcal/mol to the TLR3 and TLR4 complexes, respectively. In contrast, the contribution of hydrogen-bond energy was comparatively smaller in both systems, with values of −0.99 kcal/mol for TLR3 and  9.70 kcal/mol for TLR4. Overall, the MM-GBSA energy decomposition analysis indicated that electrostatic, van der Waals, and lipophilic interactions represented the major energetic contributors in both complexes, with larger negative contributions observed for the vaccine–TLR3 complex.

**Table 9:** MM-GBSA binding free energy of vaccine–TLR3 and vaccine–TLR4 complexes.

Parameter	Vaccine-TLR3 (Kcal/mol)	Vaccine-TLR4 (Kcal/mol)
ΔG_bind	−203.29	−55.28
Coulomb energy	−212.15	−92.74
Hydrogen bond energy	−0.99	−9.70
Lipophilic energy	−89.99	−6.46
van der Waals energy	−166.96	−43.65

#### Immune simulation

**Fig. 12:**
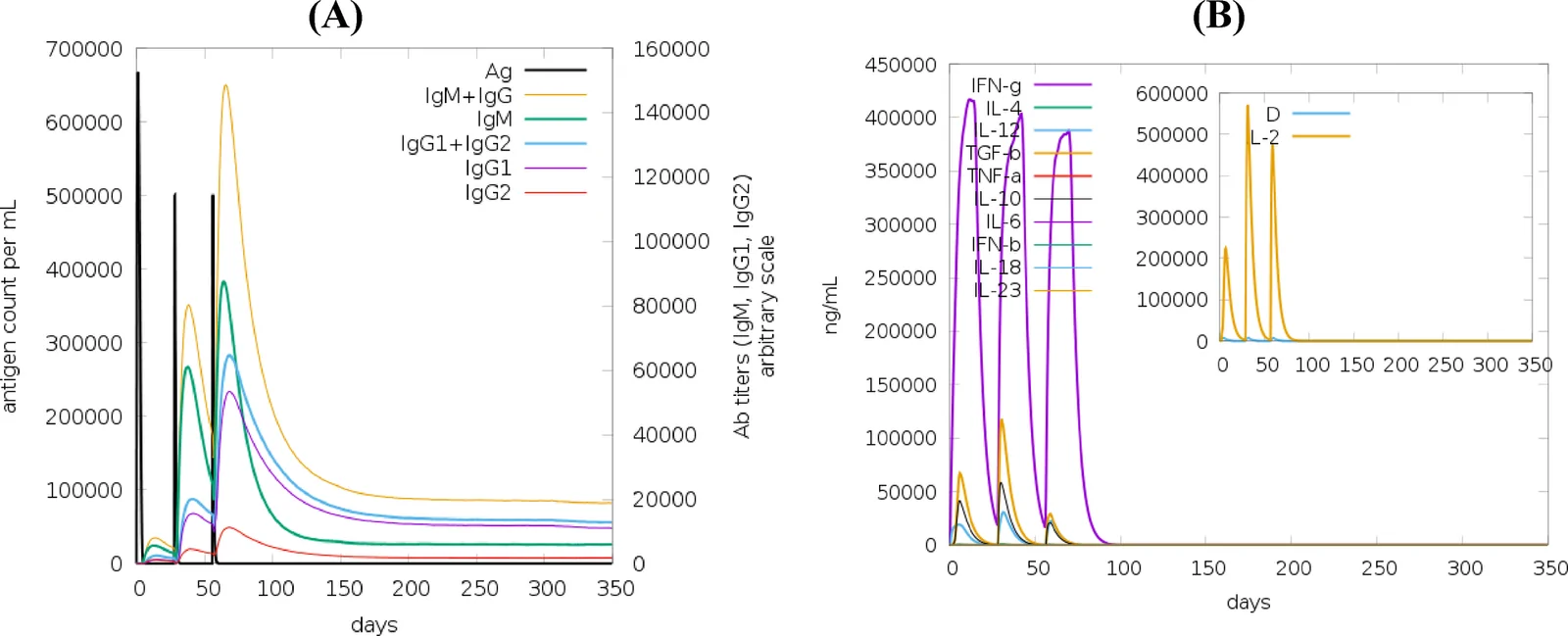
In silico immune simulation of the multi-epitope vaccine construct over 350 days using a three-dose prime–boost–boost regimen showing **(A)** antibody response dynamics and **(B)** cytokine and interleukin responses.

The immune response of the designed vaccine construct was evaluated using in silico simulation over a period of 350 days following a three-dose regimen. The antibody response profile (Fig. 12A) showed an initial increase in antigen levels followed by a corresponding rise in antibody titers after each administered dose. Elevated levels of IgM and IgG antibodies were observed following antigen exposure, with subsequent doses producing higher antibody levels than those observed after the primary dose. Different immunoglobulin subclasses, including IgG1 and IgG2, also exhibited increased levels during the simulation period. The cytokine and interleukin response (Fig. 12B) demonstrated temporal variations in the levels of immune signaling molecules. Peaks in cytokine concentrations were observed following antigen exposure, with interleukins such as IL-2 and IFN-γ exhibiting noticeable increases at specific time points during the simulation.

**Fig. 13:**
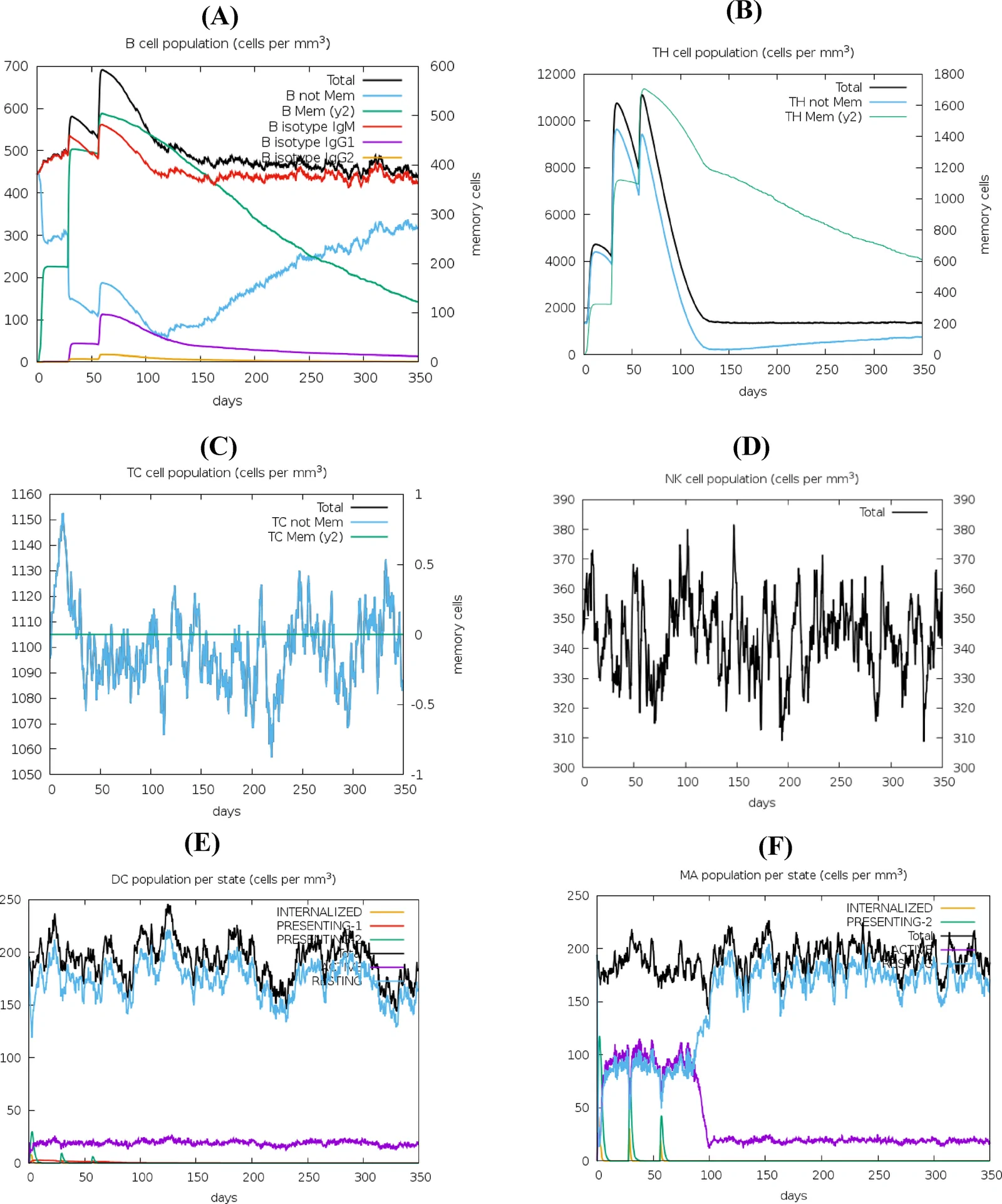
**(A)** B lymphocytes, **(B)** CD4 T-helper lymphocytes, **(C)** CD8 T-cytotoxic lymphocytes, **(D)** NK cells, **(E)** dendritic cells, and **(F)** macrophages.

The cellular immune response of the vaccine construct was also evaluated throughout the simulation period. The B-cell population (Fig. 13A) showed an initial increase following antigen exposure, with the total B-cell count reaching a peak during the early phase of the simulation. The B-memory cell population (y2) exhibited a gradual increase over time and remained detectable throughout the simulation period. In addition, the levels of different B-cell isotypes, including IgM, IgG1, and IgG2, varied over time, with IgM appearing earlier and IgG subclasses showing changes at later time points. The TH-cell population (Fig. 13B) displayed a marked increase shortly after antigen administration, followed by a decline and stabilization at lower levels over time. The TH-memory cell population (y2) remained present throughout the simulation period, with a gradual decrease after the initial peak. The TC-cell population (Fig. 13C) exhibited fluctuations throughout the simulation, with variations observed in both total and non-memory cell populations. The memory TC-cell component (y2) remained relatively stable, with limited variation across the simulation period. The NK-cell population (Fig. 13D) remained relatively consistent, showing minor fluctuations around a stable baseline throughout the simulation. The dendritic-cell population (Fig. 13E) demonstrated variation across different functional states, including internalized, presenting, and resting states. The resting dendritic-cell population constituted the major proportion, whereas internalized and presenting states showed smaller transient changes following antigen exposure. Similarly, macrophage populations (Fig. 13F) showed variation across different states, including internalized, presenting, and activated forms. The activated macrophage population exhibited changes during the early phase of the simulation, followed by stabilization over time, whereas resting macrophages remained the dominant population.

**Codon optimization and in silicoin silico cloning**

**Fig. 14:**
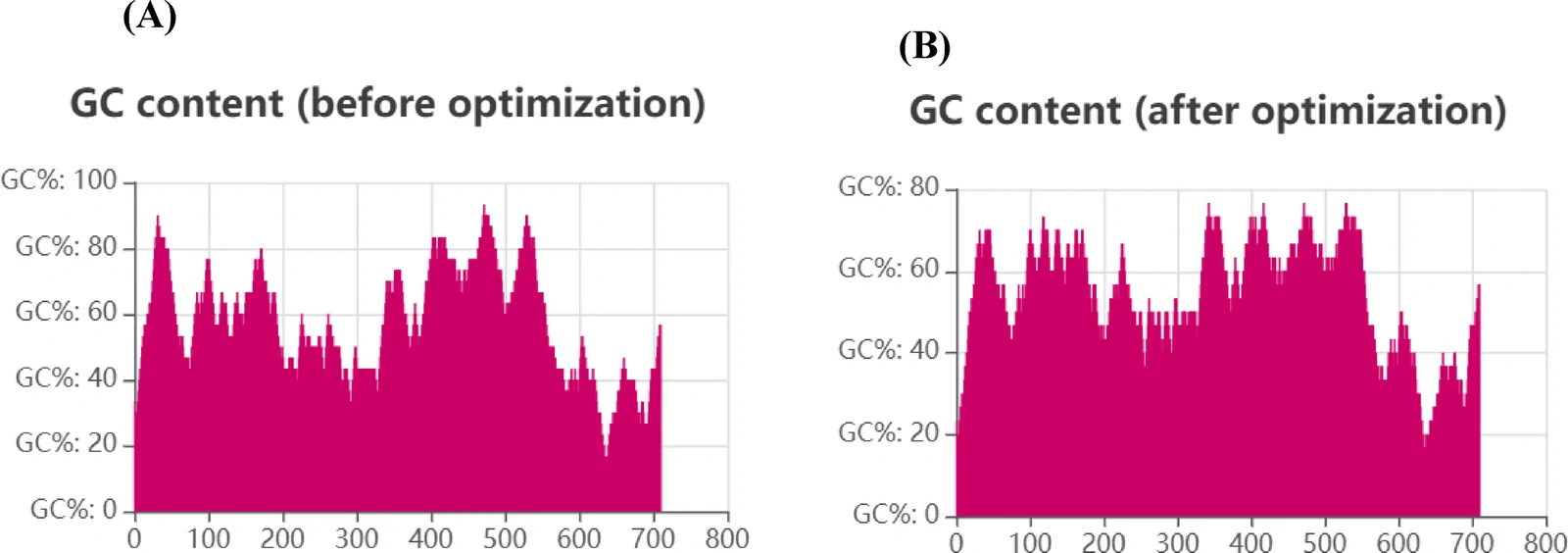
Codon optimization analysis of the designed multi-epitope vaccine construct. **(A)** GC content distribution before codon optimization. **(B)** GC content distribution after codon optimization for *Escherichia coli* K12 expression.

**Fig. 15:**
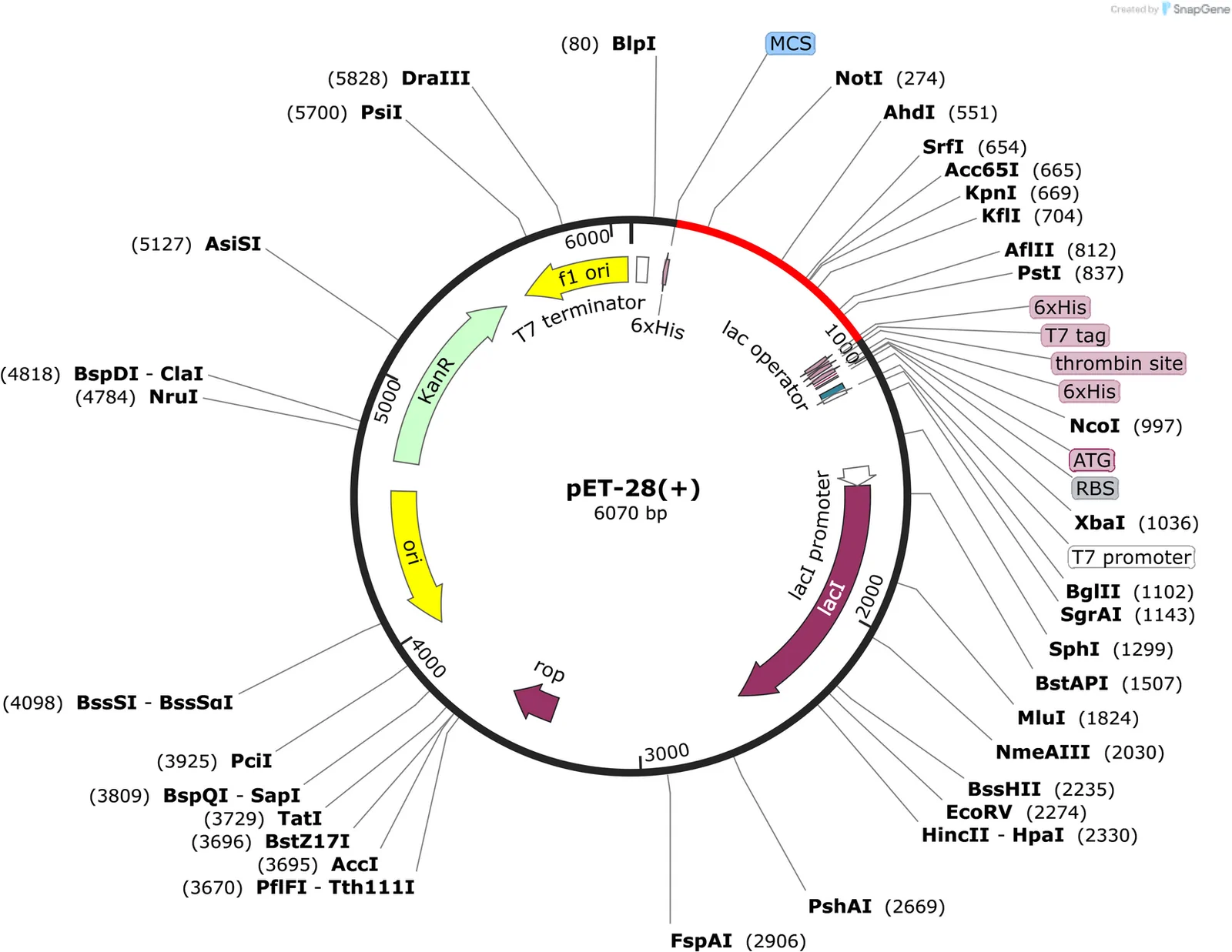
In silico cloning of the codon-optimized vaccine gene into the pET28a(+) expression vector using NdeI and XhoI restriction sites.

To evaluate the feasibility of heterologous expression, the vaccine construct was reverse-translated and subjected to codon optimization for *Escherichia coli* K12. The GC content was reduced from 56.77% before optimization to 53.65% after optimization (Fig. 14). In addition, the codon adaptation index (CAI) of the optimized sequence was predicted to be 0.75, indicating compatibility with the codon usage preferences of the bacterial host. For in silico cloning, the optimized gene sequence was inserted into the pET28a(+) expression vector using NdeI and XhoI restriction sites. The cloning simulation confirmed integration of the vaccine construct into the expression cassette with the correct orientation and maintenance of the reading frame (Fig. 15). These findings support the feasibility of recombinant expression of the designed vaccine construct in an *Escherichia coli* expression system.

## Discussion

The increasing burden of tick-borne and zoonotic viral infections has accelerated the need for rapid and targeted vaccine design strategies. In this study, a multi-epitope vaccine was designed using immunoinformatics and reverse vaccinology approaches, which are increasingly being adopted as efficient alternatives to conventional vaccine development. These approaches allow precise selection of immunogenic regions while avoiding unnecessary antigenic load and reducing potential adverse effects. The selection of cytotoxic T lymphocyte, helper T lymphocyte, and B-cell epitopes in this study was based on antigenicity, non-allergenicity, and non-toxicity. Only epitopes with antigenicity values above the standard threshold were selected to maximize their predicted immunogenic potential. A similar strategy was applied in the vaccinomics-based study on tick-borne encephalitis virus, where immunodominant epitopes were identified from the viral polyprotein to construct a targeted vaccine candidate^46^In addition, population coverage analyses reported in similar immunoinformatics-based vaccine studies often indicate broad HLA representation across diverse populations, supporting the potential applicability of multi-epitope vaccine constructs in genetically heterogeneous populations.

The use of standard linkers such as AAY, GPGPG, and KK, along with β-defensin-3 as an adjuvant, helped maintain structural integrity and improve immune stimulation. This design strategy is consistent with previous work on Kyasanur forest disease virus, where similar linkers were used to preserve epitope functionality and enhance immune responses^47^. These conserved design elements highlight the reliability of current immunoinformatics pipelines in vaccine construction. Physicochemical analysis showed that the designed vaccine is stable, with an instability index below forty, indicating structural stability. The negative grand average of hydropathicity value suggests that the protein is hydrophilic, which supports better interaction with immune cells. Comparable stability profiles have been reported in studies focusing on tick-borne viruses, where hydrophilic and stable constructs demonstrated improved immunogenic performance^48^. Structural modeling and validation confirmed the reliability of the vaccine construct, with most residues located in favorable regions of the Ramachandran plot and acceptable Z-score values. Similar structural quality has been observed in multi-epitope vaccine designs against Crimean-Congo hemorrhagic fever virus, where structural validation ensured proper folding and antigen presentation^49^.

Molecular docking analysis predicted favorable interactions between the vaccine construct and immune receptors such as Toll-like receptor 3 and Toll-like receptor 4. TLR3 and TLR4 were selected as primary receptors because of their established roles in antiviral innate immunity^35,50^. TLR3 was specifically included because Eyach virus belongs to the family *Spinareoviridae* and possesses a double-stranded RNA (dsRNA) genome, which is recognized by TLR3 and triggers downstream antiviral signaling pathways^1,38^. TLR4 is a surface-expressed pattern recognition receptor that plays an important role in the initiation of innate and adaptive immune responses^50^ and has been widely utilized in immunoinformatics-based multi-epitope vaccine studies to evaluate the immunostimulatory potential of vaccine constructs^51,52^. Furthermore, the designed vaccine incorporates β-defensin-3 as an adjuvant, which has been associated with the activation of innate immune responses through Toll-like receptor-mediated pathways^53,54^. Although other RNA-sensing receptors, such as TLR7, TLR8, and RIG-I, are also involved in antiviral immunity, they are primarily localized within endosomal compartments or the cytoplasm, where they detect viral nucleic acids following cellular entry^55,56^. In contrast, TLR4 is readily accessible on the cell surface, while TLR3 is a well-characterized sensor of viral dsRNA^38,50^. Therefore, evaluating interactions with TLR3 and TLR4 provides insight into the potential of the vaccine construct to engage key innate immune pathways and promote antiviral immune responses. Docking scores ranged between  −1380.7 for vaccine-TLR3 complex to −1073.9 for vaccine-TLR4 complex, suggesting favorable predicted interactions between the vaccine construct and the selected receptors. Similar docking trends have been reported in studies targeting Rift Valley fever virus, where favorable predicted interactions between vaccine constructs and immune receptors were observed^57^. It should be noted that docking energies were interpreted comparatively because no universal cutoff values have been established for multi-epitope vaccine–receptor docking studies. In addition, there is currently no standardized reference control for comparing docking scores across different multi-epitope vaccine constructs, as variations in sequence length, amino acid composition, tertiary structure, and interaction interfaces can substantially influence docking energies. Therefore, the observed docking scores were evaluated comparatively within the present study and interpreted together with interaction analyses and molecular dynamics simulation results to assess the stability and strength of the vaccine–receptor complexes.

Molecular dynamics simulations were further performed to evaluate the stability and flexibility of the vaccine–receptor complexes. RMSD values remained within an acceptable range, indicating stable structural behavior during simulation. RMSF values showed limited variation, and radius of gyration analysis confirmed proper compactness. Although relatively higher RMSD values were observed, particularly for the vaccine–TLR4 complex, these deviations likely reflect conformational adaptation and structural rearrangement of the multi-epitope vaccine construct during receptor engagement rather than complete destabilization of the complex. This behavior may be attributed, in part, to the presence of flexible linker sequences connecting individual epitopes, which can increase structural mobility during simulation. Furthermore, residue-level RMSF analysis demonstrated that the major fluctuations were primarily confined to exposed and linker-associated regions of the vaccine construct and were predominantly located outside the receptor–vaccine binding interfaces. In contrast, the receptor regions exhibited comparatively lower fluctuations, indicating that the interaction interfaces remained relatively stable throughout the simulation period. Together, these findings suggest that the observed structural deviations represent dynamic adaptation of the vaccine construct while preserving stable receptor engagement.

Comparable stability has been observed in vaccine designs against Crimean-Congo hemorrhagic fever virus, where molecular dynamics simulations confirmed consistent structural behavior^58^. Immune simulation analysis suggested the potential induction of humoral and cellular immune responses, characterized by increased levels of immunoglobulin M and immunoglobulin G, activation of helper and cytotoxic T cells, and elevated cytokines such as interferon gamma and interleukin two. Similar immune response profiles have been reported in vaccine designs targeting Yezo virus, where strong humoral and cellular responses were observed following simulation^59^. In addition, recent studies focusing on livestock pathogens such as goatpox virus have shown that multi-epitope vaccine constructs are predicted to induce antibody responses and cytokine production, supporting the applicability of this approach beyond human viral infections^60^. Similarly, the development of messenger RNA-based multi-epitope constructs against tick species has further demonstrated the flexibility of this approach in targeting both vectors and pathogens^61^.

Despite these promising results, some limitations remain. The study is based entirely on computational predictions, and experimental validation is required to confirm immunogenicity and safety. Epitope prediction tools may not fully represent immune responses in real biological systems, and factors such as host variability and immune regulation can influence vaccine effectiveness. Although the IEDB Next Generation Tools pipeline and associated prediction algorithms provide improved prediction performance, epitope selection remains dependent on computational models and available training datasets, which may result in false-positive or false-negative predictions. In addition, the cytokine-inducing potential of the selected HTL epitopes, including IFN-γ, IL-4, and IL-10 induction, was not evaluated in the present study because the relevant prediction servers were inaccessible during this study. Consequently, the ability of the selected HTL epitopes to stimulate specific cytokine responses should be evaluated in future computational and experimental studies. Similarly, the tertiary structure of the vaccine construct was generated using AlphaFold3, which provides highly accurate structural predictions; however, predicted models cannot fully replace experimentally resolved structures obtained through X-ray crystallography, NMR spectroscopy, or cryo-electron microscopy. In addition, molecular docking analyses provide a static representation of receptor–vaccine interactions and may not completely capture the complexity of molecular recognition under physiological conditions. Although molecular dynamics simulations were performed to assess the stability and flexibility of the vaccine–receptor complexes, the results remain dependent on simulation parameters, force-field selection, and the 100 ns simulation timescale, which may not fully reflect long-term molecular behavior in vivo. These challenges are common across immunoinformatics-based vaccine studies and highlight the importance of experimental validation. Therefore, the predicted epitopes, structural model, receptor interactions, and immune response profiles should be verified through in vitro and in vivo studies, including antigen expression analyses, immunogenicity assays, and challenge experiments. Successful experimental validation of these findings would further support the potential of the proposed vaccine candidate for future development against Eyach virus.

## Conclusion

This study highlights the significance of immunoinformatics in rapidly designing a multi-epitope vaccine candidate against Eyach virus, demonstrating strong antigenicity, stability, and broad global population coverage, which underscores its potential contribution to preventing tick-borne encephalitis–like infections. The findings support computational vaccine design as a cost-effective and targeted alternative to traditional approaches. However, the study is limited by its exclusive reliance on in silico analyses, which cannot fully replicate complex biological systems, immune variability, or real-world safety and efficacy. Future prospects should focus on experimental validation, including in vitro expression, in vivo immunogenicity, and clinical studies, to confirm protective efficacy, safety, and long-term immune responses before practical application.

## Data Availability

All data generated or analyzed during this study are included in this article. Additional data including raw data associated with this study can be assessed through this link https://drive.google.com/drive/folders/100r92tezN2qp4wHpA0USLg-Hx9yv90by? usp=drive_link.

## References

[1] Matthijnssens, J. et al. ICTV Virus Taxonomy Profile: Spinareoviridae 2022. *J. Gen. Virol*. **103**, 001785 (2022). 10.1099/jgv.0.001781 PMC12642366 36394457

[2] Moutailler, S. et al. Diversity of viruses in Ixodes ricinus, and characterization of a neurotropic strain of Eyach virus. *New Microbes New Infect*. **11**, 71–81 (2016). 27158509 10.1016/j.nmni.2016.02.012 PMC4845080

[3] Calisher, C. H. Medically important arboviruses of the United States and Canada. *Clin. Microbiol. Rev.* **7** (1), 89–116 (1994). 8118792 10.1128/cmr.7.1.89 PMC358307

[4] Rochlin, I. & Toledo, A. Emerging tick-borne pathogens of public health importance: a mini-review. *J. Med. Microbiol.* **69** (6), 781–791 (2020). 32478654 10.1099/jmm.0.001206 PMC7451033

[5] Hart, C. E. & Thangamani, S. Tick-virus interactions: current understanding and future perspectives. *Parasite Immunol.* **43** (5), e12815 (2021). 33368375 10.1111/pim.12815

[6] İnci, A. et al. A Compendium Review of the Global Epidemiology of Ticks and Tick-borne Diseases: Regional Insights from Türkiye. *Turkiye Parazitol Derg*. **49** (Suppl 1), 1–66 (2025). 41498249 10.4274/tpd.galenos.2025.82713

[7] Chiffi, G. et al. Tick-borne encephalitis: A comprehensive review of the epidemiology, virology, and clinical picture. *Rev. Med. Virol.* **33** (5), e2470 (2023). 37392370 10.1002/rmv.2470

[8] Deshpande, G. et al. Assessing the influence of climate change and environmental factors on the top tick-borne diseases in the United States: a systematic review. *Microorganisms* **12** (1), 50 (2023). 38257877 10.3390/microorganisms12010050 PMC10821204

[9] Rupani, A., Elshabrawy, H. A. & Bechelli, J. Dermatological manifestations of tick-borne viral infections found in the United States. *Virol. J.* **19** (1), 199 (2022). 36443864 10.1186/s12985-022-01924-w PMC9702624

[10] Ye, R. Z. et al. Tick-borne viruses: discovery, clinical aspects, influencing factors and control. *Nat. Rev. Microbiol. *1–19. (2026). 10.1038/s41579-026-01277-z 41555040

[11] Hromníková, D. et al. Prevention of tick-borne diseases: challenge to recent medicine. *Biologia* **77** (6), 1533–1554 (2022). 35283489 10.1007/s11756-021-00966-9 PMC8905283

[12] Saraswathi, A. et al. Vaccines as preventive measures, in Drug Discovery and One Health Approach in Combating Infectious Diseases (eds. Pandey, K. B., Newman, D. J. & Egbuna, C.). Elsevier. 205–235. (2025).

[13] Kang, S. M. & Compans, R. W. Host responses from innate to adaptive immunity after vaccination: molecular and cellular events. *Mol. Cells*. **27** (1), 5–14 (2009). 19214429 10.1007/s10059-009-0015-1 PMC6280669

[14] Palma, M. Epitopes and mimotopes identification using phage display for vaccine development against infectious pathogens. *Vaccines* **11** (7), 1176 (2023). 37514992 10.3390/vaccines11071176 PMC10384025

[15] Wei, Y. et al. Advances of computational methods enhance the development of multi-epitope vaccines. *Brief. Bioinform.* **26** (1), bbaf055 (2025). 10.1093/bib/bbaf055 PMC11827616 39951549

[16] Ananya et al. Vaccine design and development: exploring the interface with computational biology and AI. *Int. Rev. Immunol.* **43** (6), 361–380 (2024). 38982912 10.1080/08830185.2024.2374546

[17] Cernuto, F. et al. In-silico epitope-based vaccines design: progress, challenges and the road ahead. *Expert Opin. Drug Discov.* **20** (12), 1701–1712 (2025). 41354625 10.1080/17460441.2025.2599178

[18] Krogh, A. et al. Predicting transmembrane protein topology with a hidden Markov model: application to complete genomes. *J. Mol. Biol.* **305** (3), 567–580 (2001). 11152613 10.1006/jmbi.2000.4315

[19] Doneva, N. & Dimitrov, I. Viral immunogenicity prediction by machine learning methods. *Int. J. Mol. Sci.* **25** (5), 2949 (2024). 38474194 10.3390/ijms25052949 PMC10932092

[20] Dimitrov, I. et al. AllerTOP v. 2—a server for in silico prediction of allergens. *J. Mol. Model.* **20** (6), 2278 (2014). 24878803 10.1007/s00894-014-2278-5

[21] Reynisson, B. et al. NetMHCpan-4.1 and NetMHCIIpan-4.0: improved predictions of MHC antigen presentation by concurrent motif deconvolution and integration of MS MHC eluted ligand data. *Nucleic Acids Res.* **48** (W1), W449–w454 (2020). 32406916 10.1093/nar/gkaa379 PMC7319546

[22] Kaabinejadian, S. et al. Accurate MHC Motif Deconvolution of Immunopeptidomics Data Reveals a Significant Contribution of DRB3, 4 and 5 to the Total DR Immunopeptidome. *Front. Immunol.* **13**, 835454 (2022). 35154160 10.3389/fimmu.2022.835454 PMC8826445

[23] Bui, H. H. et al. Development of an epitope conservancy analysis tool to facilitate the design of epitope-based diagnostics and vaccines. *BMC Bioinform.* **8**, 361 (2007). 10.1186/1471-2105-8-361 PMC2233646 17897458

[24] Bui, H. H. et al. Predicting population coverage of T-cell epitope-based diagnostics and vaccines. *BMC Bioinform.* **7**, 153 (2006). 10.1186/1471-2105-7-153 PMC1513259 16545123

[25] Asim, M. & Makki, H. H. Molecular characterization and immunoinformatics-based design of a multi-epitope vaccine against Staphylococcus nepalensis. *Antonie van Leeuwenhoek*. **119** (5), 91 (2026). 41949632 10.1007/s10482-026-02303-z

[26] Wilkins, M. R. et al. Protein identification and analysis tools in the ExPASy server. *Methods Mol. Biol.* **112**, 531–552 (1999). 10027275 10.1385/1-59259-584-7:531

[27] Hanif, N. et al. In silico characterization of hypothetical protein AZJ53_10480 in Streptococcus pneumoniae. *BioScientific Rev.* **6** (4), 1–12 (2024).

[28] Hon, J. et al. SoluProt: prediction of soluble protein expression in Escherichia coli. *Bioinformatics* **37** (1), 23–28 (2021). 33416864 10.1093/bioinformatics/btaa1102 PMC8034534

[29] Abramson, J. et al. Accurate structure prediction of biomolecular interactions with AlphaFold 3. *Nature* **630** (8016), 493–500 (2024). 38718835 10.1038/s41586-024-07487-w PMC11168924

[30] Ko, J. et al. GalaxyWEB server for protein structure prediction and refinement. *Nucleic Acids Res.* **40** (W1), W294–W297 (2012). 22649060 10.1093/nar/gks493 PMC3394311

[31] Hanif, N. et al. Computational drug design targeting MYH7 for hypertrophic cardiomyopathy integrating molecular docking, Density Functional Theory, and Molecular Dynamics Simulations. *J. Appl. Biol. Sci.* **19** (3), 179–192 (2025).

[32] Wiederstein, M. & Sippl, M. J. ProSA-web: interactive web service for the recognition of errors in three-dimensional structures of proteins. *Nucleic Acids Res*. **35**, W407–W410 (2007). 17517781 10.1093/nar/gkm290 PMC1933241

[33] Ponomarenko, J. et al. ElliPro: a new structure-based tool for the prediction of antibody epitopes. *BMC Bioinform.* **9**, 514 (2008). 10.1186/1471-2105-9-514 PMC2607291 19055730

[34] Kozakov, D. et al. The ClusPro web server for protein–protein docking. *Nat. Protoc.* **12** (2), 255–278 (2017). 28079879 10.1038/nprot.2016.169 PMC5540229

[35] Lester, S. N. & Li, K. Toll-like receptors in antiviral innate immunity. *J. Mol. Biol.* **426** (6), 1246–1264 (2014). 24316048 10.1016/j.jmb.2013.11.024 PMC3943763

[36] Duan, T. et al. Toll-like receptor signaling and its role in cell-mediated immunity. *Front. Immunol.* **13**, 812774 (2022). 35309296 10.3389/fimmu.2022.812774 PMC8927970

[37] Thompson, M. R. et al. Pattern recognition receptors and the innate immune response to viral infection. *Viruses* **3** (6), 920 (2011). 21994762 10.3390/v3060920 PMC3186011

[38] Schröder, M. & Bowie, A. G. TLR3 in antiviral immunity: key player or bystander? *Trends Immunol*. **26**(9), 462–468. (2005). 10.1016/j.it.2005.07.002 16027039

[39] Werling, D. & Jungi, T. W. TOLL-like receptors linking innate and adaptive immune response. *Vet. Immunol. Immunopathol.* **91** (1), 1–12 (2003). 12507844 10.1016/s0165-2427(02)00228-3

[40] Laskowski, R. A. PDBsum 1: A standalone program for generating PDBsum analyses. *Protein Sci.* **31** (12), e4473 (2022). 36251626 10.1002/pro.4473 PMC9667822

[41] Bowers, K. J. et al. Scalable algorithms for molecular dynamics simulations on commodity clusters. in Proceedings of the 2006 ACM/IEEE Conference on Supercomputing. (2006).

[42] Hildebrand, P. W., Rose, A. S. & Tiemann, J. K. Bringing molecular dynamics simulation data into view. *Trends Biochem. Sci*. **44**(11), 902–913 (2019). 10.1016/j.tibs.2019.06.004 31301982

[43] Shivakumar, D. et al. Prediction of absolute solvation free energies using molecular dynamics free energy perturbation and the OPLS force field.* J. Chem.Theory Comput*. **6**(5), 1509–1519. (2010). 10.1021/ct900587b 26615687

[44] Laubenbacher, R. et al. Toward mechanistic medical digital twins: some use cases in immunology. *Front. Digit. Health*. **6**, 1349595 (2024). 38515550 10.3389/fdgth.2024.1349595 PMC10955144

[45] Madeira, F. et al. The EMBL-EBI Job Dispatcher sequence analysis tools framework in 2024. *Nucleic Acids Res.* **52** (W1), W521–W525 (2024). 38597606 10.1093/nar/gkae241 PMC11223882

[46] Rahman, S. et al. Vaccinomics-based identification of immunodominant epitopes in the tick-borne encephalitis virus polyprotein for multi-epitope vaccine development. *Comput. Struct. Biotechnol. Rep.* **2**, 100047 (2025).

[47] Adla, D. et al. Immunoinformatic-based multi-epitope vaccine design and validation against Kyasanur forest disease: A tick-borne viral infection. *J. Vector Borne Dis.* **62** (3), 369–379 (2025). 39936178 10.4103/jvbd.jvbd_84_24

[48] Ben Said, M. & Kratou, M. Exploring approaches and advancements in the development and evaluation of multi-epitope subunit vaccines against tick-borne viruses. *Vet. Microbiol.* **306**, 110577 (2025). 40466409 10.1016/j.vetmic.2025.110577

[49] Shrivastava, N., Verma, A. & Dash, P. K. Identification of functional epitopes of structural proteins and in-silico designing of dual acting multiepitope anti-tick vaccine against emerging Crimean-Congo hemorrhagic fever virus. *Eur. J. Pharm. Sci.* **151**, 105396 (2020). 32479862 10.1016/j.ejps.2020.105396

[50] Akira, S. & Takeda, K. Toll-like receptor signalling. *Nat. Rev. Immunol.* **4** (7), 499–511 (2004). 15229469 10.1038/nri1391

[51] Waqas, M. et al. Immunoinformatics design of multivalent epitope vaccine against monkey poxvirus and its variants using membrane-bound, enveloped, and extracellular proteins as targets. *Front. Immunol.* **14**, 1091941 (2023). 36776835 10.3389/fimmu.2023.1091941 PMC9908764

[52] Alam, R. et al. In silico formulation of a next-generation multiepitope vaccine for use as a prophylactic candidate against Crimean-Congo hemorrhagic fever. *BMC Med.* **21** (1), 36 (2023). 36726141 10.1186/s12916-023-02750-9 PMC9891764

[53] Funderburg, N. et al. Human β-defensin-3 activates professional antigen-presenting cells via Toll-like receptors 1 and 2. *Proc. Natl. Acad. Sci.* **104** (47), 18631–18635 (2007). 18006661 10.1073/PNAS.0702130104 PMC2141828

[54] Biragyn, A. et al. Toll-like receptor 4-dependent activation of dendritic cells by β-defensin 2. *Science* **298** (5595), 1025–1029 (2002). 12411706 10.1126/science.1075565

[55] Kawai, T. & Akira, S. The role of pattern-recognition receptors in innate immunity: update on Toll-like receptors. *Nat. Immunol.* **11** (5), 373–384 (2010). 20404851 10.1038/ni.1863

[56] Reikine, S., Nguyen, J. B. & Modis, Y. Pattern recognition and signaling mechanisms of RIG-I and MDA5. *Front. Immunol.* **5**, 342 (2014). 25101084 10.3389/fimmu.2014.00342 PMC4107945

[57] Fatima, I. et al. Designing of a multi-epitopes-based peptide vaccine against rift valley fever virus and its validation through integrated computational approaches. *Comput. Biol. Med.* **141**, 105151 (2022). 34942394 10.1016/j.compbiomed.2021.105151

[58] Tahir Ul Qamar, M. et al. Development of a Novel Multi-Epitope Vaccine Against Crimean-Congo Hemorrhagic Fever Virus: An Integrated Reverse Vaccinology, Vaccine Informatics and Biophysics Approach. *Front. Immunol.* **12**, 669812 (2021). 34220816 10.3389/fimmu.2021.669812 PMC8242340

[59] Ayaz, H. et al. Molecular modeling to design a multiepitope vaccine against emerging tick-borne Yezo virus and its validation through biophysics techniques. *Silico Pharmacol.* **13** (2), 80 (2025). 10.1007/s40203-025-00370-0 PMC12141707 40487341

[60] Long, Q. et al. Design of a multi-epitope vaccine against goatpox virus using an immunoinformatics approach. *Front. Cell. Infect. Microbiol.* **13**, 1309096 (2023). 38487680 10.3389/fcimb.2023.1309096 PMC10937444

[61] Ullah, U. et al. Designing a multi-epitope construct using immuno-informatic tools to prepare a messenger RNA vaccine against Rhipicephalus microplus ticks. *Vet. World*. **17** (10), 2235–2247 (2024). 39619941 10.14202/vetworld.2024.2235-2247 PMC11606274

